# 8(*meso*)-Pyridyl-BODIPYs: Effects of 2,6-Substitution with Electron-Withdrawing Nitro, Chloro, and Methoxycarbonyl Groups

**DOI:** 10.3390/molecules28124581

**Published:** 2023-06-06

**Authors:** Caroline Ndung’U, Petia Bobadova-Parvanova, Daniel J. LaMaster, Dylan Goliber, Frank R. Fronczek, Maria da Graça H. Vicente

**Affiliations:** 1 Department of Chemistry, Louisiana State University, Baton Rouge, LA 70803, USA; cndung2@lsu.edu (C.N.); daniel.j.lamaster@gmail.com (D.J.L.); ffroncz@lsu.edu (F.R.F.); 2 Department of Chemistry and Fermentation Sciences, Appalachian State University, Boone, NC 28608, USA; bobadovap@appstate.edu (P.B.-P.); goliberda@appstate.edu (D.G.)

**Keywords:** BODIPY, pyridyl, nitro, chloro, methyl ester

## Abstract

The introduction of electron-withdrawing groups on 8(*meso*)-pyridyl-BODIPYs tends to increase the fluorescence quantum yields of this type of compound due to the decrease in electronic charge density on the BODIPY core. A new series of 8(*meso*)-pyridyl-BODIPYs bearing a 2-, 3-, or 4-pyridyl group was synthesized and functionalized with nitro and chlorine groups at the 2,6-positions. The 2,6-methoxycarbonyl-8-pyridyl-BODIPYs analogs were also synthesized by condensation of 2,4-dimethyl-3-methoxycarbonyl-pyrrole with 2-, 3-, or 4-formylpyridine followed by oxidation and boron complexation. The structures and spectroscopic properties of the new series of 8(*meso*)-pyridyl-BODIPYs were investigated both experimentally and computationally. The BODIPYs bearing 2,6-methoxycarbonyl groups showed enhanced relative fluorescence quantum yields in polar organic solvents due to their electron-withdrawing effect. However, the introduction of a single nitro group significantly quenched the fluorescence of the BODIPYs and caused hypsochromic shifts in the absorption and emission bands. The introduction of a chloro substituent partially restored the fluorescence of the mono-nitro-BODIPYs and induced significant bathochromic shifts.

## 1. Introduction

Over the last three decades, boron dipyrromethene, abbreviated BODIPY, dyes have been synthesized and investigated [1,2,3] for a wide range of applications. These include their use as photosensitizers for photodynamic therapy, as biological labels, as imaging agents [4,5], as fluorescence switches, and as laser dyes [6,7,8]. These diverse BODIPY applications are due to their remarkable properties, which include large molar absorption coefficients [7,8,9,10], high photochemical stability, narrow spectral band widths, generally high fluorescence quantum yields, and low cytotoxicity [1,2,3]. In addition, due to their tunable properties, BODIPY applications have continued to attract researchers’ attention.

BODIPYs are easily functionalized at all the carbon atoms and at the boron center, enabling the fine tuning of their chemical and photophysical properties for a particular application [11,12]. In particular, the introduction of water-solubilizing groups, including phosphates [13], sulfonates [14], carboxylates [15], carbohydrates [16], and oligoethylene glycol chains [17], has allowed the development of water-soluble BODIPYs for biological applications. Among the cationic BODIPY derivatives, 8(*meso*)-pyridyl-substituted BODIPYs have received special attention. This is due to their ability to coordinate with metals and the easy protonation or alkylation of the pyridyl groups, which has led to their use as pH sensors [18,19], as mitochondria-specific probes [20,21], as photosensitizers for antimicrobial photodynamic inactivation [22,23], as metal ion sensors [8,24], as G-series nerve agent sensors [25], and as photocatalysts for hydrogen production [26,27]. We have recently reported that the functionalization of 8(*meso*)-pyridyl-BODIPYs with moderate electron-withdrawing chlorine atoms at the 2,6-positions induces bathochromic shifts in the absorption and emission wavelengths of the resulting BODIPYs, increases their reduction potentials, and enhances their relative fluorescence quantum yields [28,29]. We hypothesized that stronger electron-withdrawing groups at the 2,6-positions of 8(*meso*)-pyridyl-BODIPYs would have stronger effects on their photophysical and electrochemical properties. Furthermore, mono-2-functionalized BODIPY derivatives and the introduction of two different electron-withdrawing groups at the 2 and 6 positions induce molecular asymmetry and can, therefore, also affect their properties. Herein, we report the synthesis and investigation of 8(*meso*)-pyridyl-BODIPYs bearing electron-withdrawing 2,6-methylester substituents and the asymmetric 2-nitro and 2-chloro-6-nitro BODIPY derivatives. While the nitro and chlorine groups can be directly introduced by functionalization of the BODIPY via electrophilic substitution, the 2,6-methoxycarbonyl-8-pyridyl-BODIPYs were prepared from 2,4-dimethyl-3-methoxycarbonyl-pyrrole and the corresponding pyridine carboxaldehyde. The presence of the methyl groups at the 1,7-positions of these 8(*meso*)-pyridyl-BODIPYs prevents the rotation of the *meso*-pyridyl groups, enhancing their fluorescence properties and inducing nearly perpendicular dihedral angles between the pyridyl group and the BODIPY core [11,27,28].

## 2. Results and Discussion

### 2.1. Synthesis

**Scheme 1:**
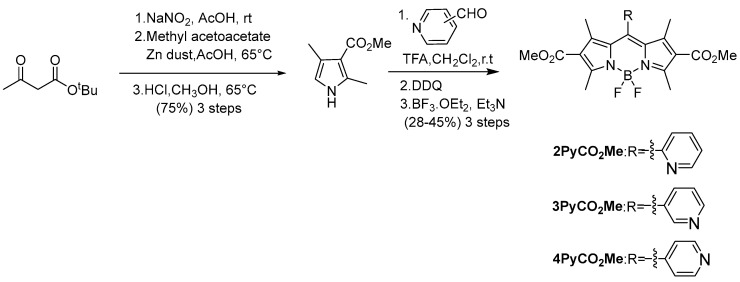
Synthesis of **2PyCO_2_Me**, **3PyCO_2_Me**, and **4PyCO_2_Me**.

The syntheses of **2PyCO_2_Me**, **3PyCO_2_Me**, and **4PyCO_2_Me** were accomplished as shown in Scheme 1. The 2,4-dimethyl-3-methoxycarbonyl-pyrrole was prepared from *tert*-butyl acetoacetate and methyl acetoacetate in three steps using the Knorr pyrrole synthesis [30]. The condensation of 2,4-dimethyl-3-methoxycarbonyl-pyrrole with 2-, 3-, or 4-formylpyridine in the presence of TFA followed by oxidation with DDQ (2,3-dichloro-5,6-dicyano-1,4-benzoquinone) and boron complexation using boron trifluoride diethyl etherate produced the corresponding functionalized BODIPYs in 45%, 40%, and 28% yields, respectively. The lower yield obtained for **4PyCO_2_Me** is due to its lower stability on silica gel, which made its purification with chromatography more challenging. The structures of the three isomers were confirmed with ^1^H-NMR and X-ray crystallography. Interestingly, these molecules can adopt different conformations depending on the orientation of the carbonyl groups on the methyl esters relative to the BODIPY core (see Section 2.2).

**Scheme 2:**
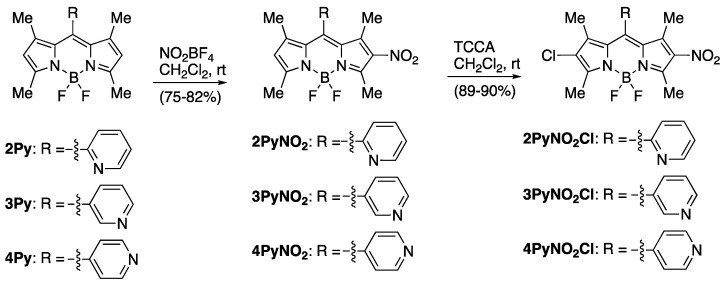
Direct functionalization of **2Py**, **3Py**, and **4Py** with nitration and chlorination.

The mono-nitrated derivatives **2PyNO_2_**, **3PyNO_2_**, and **4PyNO_2_** were obtained by nitration of the corresponding 8(*meso*)-pyridyl-BODIPYs using NO_2_BF_4_ in dichloroethane [31] in 82%, 82%, and 77% yield, respectively, as shown in Scheme 2. The single pyrrolic proton of these BODIPY derivatives was clearly observed in the ^1^H-NMR spectrum at approximately 6.5 ppm (see Supplemental Materials). Chlorination of these three compounds using trichloroisocyanuric acid (TCCA) in dichloromethane [28,29] gave the corresponding **2PyNO_2_Cl**, **3PyNO_2_Cl**, and **4PyNO_2_Cl** BODIPY derivatives in nearly quantitative yields. The structures of all BODIPYs were confirmed with ^1^H, ^13^C NMR, HRMS, and X-ray crystallography (see Supplemental Materials).

### 2.2. X-ray and Computational Structural Analysis

**Figure 1:**
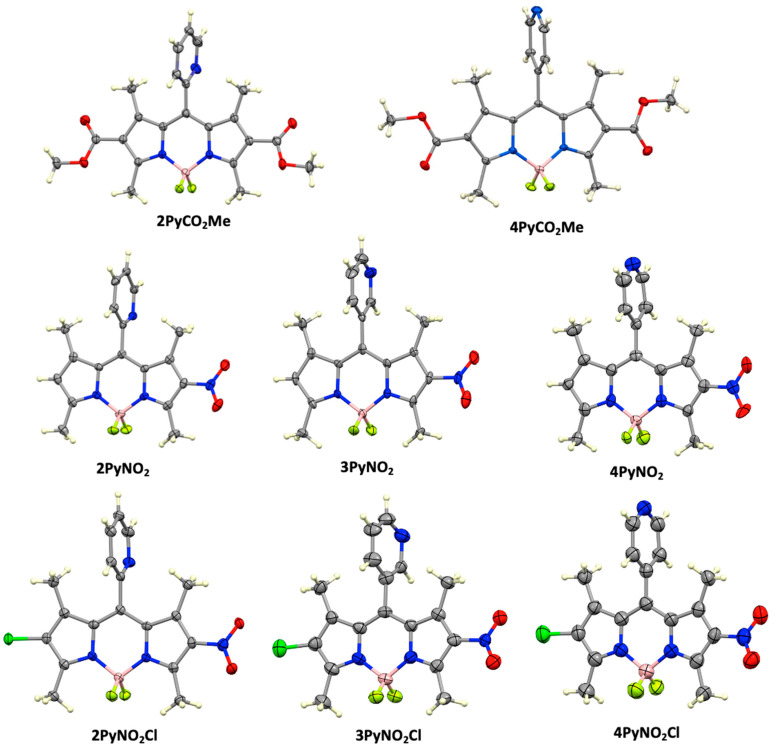
Crystal structures of **2PyCO_2_Me**, **4PyCO_2_Me**, **2PyNO_2_**, **3PyNO_2_**, **4PyNO_2_**, **2PyNO_2_Cl**, **3PyNO_2_Cl**, and **4PyNO_2_Cl** with 50% ellipsoids.

Crystals suitable for X-ray analysis were obtained for **2PyCO_2_Me**, **4PyCO_2_Me**, **2PyNO_2_**, **3PyNO_2_**, **4PyNO_2_**, **2PyNO_2_Cl**, **3PyNO_2_Cl**, and **4PyNO_2_Cl** with slow evaporation of dichloromethane and hexanes. The structures are shown in Figure 1. For **2PyCO_2_Me**, the 12-atom BODIPY core is fairly planar with a mean deviation of 0.029 Å. The 2-pyridyl substituent is disordered by a 180° rotation, switching the 2 and 6 positions. Only one orientation is shown in Figure 1. The 2-pyridyl and the BODIPY core planes form a dihedral angle of 84.7°, and the two CO_2_Me planes form dihedral angles of 16.0° and 22.1° with the BODIPY core. For the **4PyCO_2_Me**, the central six-membered C_3_N_2_B ring has an envelope distortion from planarity with the B atom lying 0.133 Å out of the plane of the other five atoms. The 12-atom BODIPY core thus has a bowed conformation with the C atoms at the 2 and 6 positions carrying the ester substituents lying 0.127 Å and 0.128 Å out of the core plane both on the opposite side from the B atom. The 4-pyridyl ring forms a dihedral angle of 84.0° with the BODIPY core, and the CO_2_Me planes form dihedral angles of 8.7° and 11.7° with the BODIPY core. The C=O groups are nearly in the planes of their respective pyrroles with the O atoms lying only 0.023 Å and 0.045 Å out of the planes.

The performed computational studies are in agreement with the above experimental findings. We investigated three different conformations computationally with the two C=O bonds oriented toward the *meso*-pyridyl ring with the two C=O bonds oriented away from the *meso*-pyridyl ring and with one C=O bond toward and one away from the *meso*-pyridyl ring (Figure S1a, Supplemental Materials). The energy differences between the three possible structures are very small (between 0.1 and 0.7 kcal/mol); therefore, all of them likely exist in solution. The pyridyl rings are nearly perpendicular to the BODIPY core with dihedral angles of 86° to 87°. The two CO_2_Me groups are not co-planar with the BODIPY core, forming dihedral angles of 18° to 19° for **2PyCO_2_Me**, **3PyCO_2_Me**, and **4PyCO_2_Me**.

For the **2PyNO_2_**, the 12-atom BODIPY core is fairly planar with a mean deviation of 0.027 Å. The 2-pyridyl ring forms a dihedral angle of 84.9° with the BODIPY core, and the nitro group makes a dihedral angle of 19.6° with the BODIPY core. In the case of **3PyNO_2_**, the molecular structure is very similar to that of the 2-pyridyl isomer with a mean deviation of the 12 BODIPY core atoms of 0.015 Å, the 3-pyridyl ring having an 88.8° dihedral angle with it, and the nitro group having a 23.7° dihedral angle with it. Similarly, for the **4PyNO_2_**, the molecular structure as the hexane solvate is also similar to those of the 2- and 3-pyridyl isomers. The mean deviation from the BODIPY plane is 0.031 Å. The 4-pyridyl ring makes a dihedral angle of 86.3° with it. The nitro group appears less tilted out of the BODIPY plane, however, having a 4.4° dihedral angle with the BODIPY core. As previously observed [29], the dihedral angle of the *meso*-pyridyl group with the BODIPY core is slightly lower in the case of the 2-pyridyl compared with the 3- and 4-pyridyl analogs, although the difference is small (1–4°).

The computational modeling predicts similar structures and small differences between **2PyNO_2_**, **3PyNO_2_**, and **4PyNO_2_** (Figure S1b, Supplemental Materials). The pyridyl rings form dihedral angles of 81°, 80°, and 82°, respectively. The nitro group is oriented at 15°, 16°, and 17°, respectively. In the case of **3PyNO_2_** and **4PyNO_2_**, two possible structures were investigated, i.e., with the pyridyl nitrogen oriented away from the viewer or with the pyridyl nitrogen oriented toward the viewer. The energy differences are very small (0.1–0.2 kcal/mol); therefore, both orientations are possible in solution.

For the **2PyNO_2_Cl**, the molecule is disordered in the crystal with the nitro and chloro substituents swapped approximately 13% of the time. Only one orientation is shown in Figure 1. The 12-atom BODIPY core is fairly planar with a 0.039 Å mean deviation. The 2-pyridyl group forms a dihedral angle of 84.4° with the BODIPY core. The nitro group in this compound forms a dihedral angle of 21.0° (weighted average of two) with the BODIPY core. The structure of **3PyNO_2_Cl** has three independent molecules, two of which lie on mirror planes in the crystal. There is no disorder between the nitro and chloro groups as was observed in the 2-pyridyl isomer; however, for the mirror-symmetric molecules, the 3-pyridyl group, which lies across the mirror, is necessarily disordered. In the mirror-symmetric molecules, the BODIPY cores are planar by symmetry, the nitro groups are coplanar with the BODIPY core, and the 3-pyridyl rings are exactly perpendicular by symmetry to the BODIPY cores. For the asymmetric molecule, the BODIPY core is nearly planar with a 0.019 Å mean deviation, the 3-pyridyl ring forms a dihedral angle of 80.0° with the core, and the nitro group has a 16.1° dihedral angle with the core. Since the displacement parameters are large for this structure determination, there may be unresolved disorder that affects the above values. For the **4PyNO_2_Cl**, there are two independent molecules with nearly identical conformations with the BODIPY core being nearly planar. Averaged over the two molecules, the mean core deviation is 0.019 Å, the 4-pyridyl dihedral angle with the BODIPY core is 84.8°, and the nitro group dihedral angle is 18.6°.

Computational modelling predicts a pyridyl-BODIPY dihedral angle of 81°, 79°, and 81° for **2PyNO_2_Cl**, **3PyNO_2_Cl**, and **4PyNO_2_Cl**, respectively, and a nitro-BODIPY dihedral angle of 16° for all three molecules (Figure S1c, Supplemental Materials). The two possible orientations of the pyridyl ring (pyridyl nitrogen toward or away from the viewer) showed very small energy differences (0.1–0.2 kcal/mol) in the case of **3PyNO_2_Cl** and **4PyNO_2_Cl**; therefore, both orientations are possible in solution.

### 2.3. Spectroscopic Properties

The absorption and emission spectra of the BODIPYs were obtained at room temperature in acetonitrile, methanol, and toluene, and the results are summarized in Table 1 and  Table S1 (Supplemental Materials). The computationally modeled spectroscopic and electronic properties are given in Table 2. The results obtained for the 2,6-unsubstituted 8(*meso*)-pyridyl-BODIPYs [28] are also given for comparison purposes. All BODIPYs exhibit a characteristic strong absorption peak attributed to the S_0_-S_1_ (π-π*) transition that appears at similar maximum absorption and emission wavelength values within each individual series of pyridyl-BODIPYs. The performed calculations show that this is the dominant transition contributing approximately 70% to the total absorption or emission. The next excited singlet state is more than 0.9 eV higher in energy.

**Figure 2:**
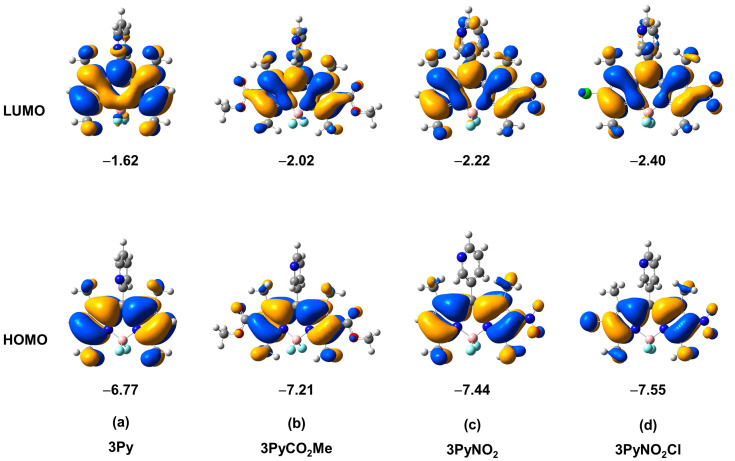
Frontier orbitals of (**a**) **3Py [29]**, (**b**) **3PyCO_2_Me**, (**c**) **3PyNO_2_**, and (**d**) **3PyNO_2_Cl** BODIPYs. Orbital energies in eV. The frontier orbitals for the entire series are given in Appendix A.

**Figure A1:**
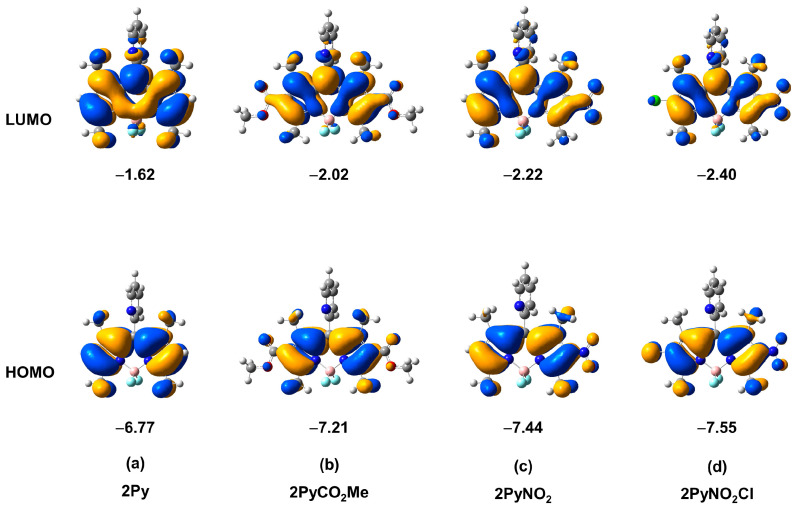
Frontier orbitals of (**a**) **2Py [29]**, (**b**) **2PyCO_2_Me**, (**c**) **2PyNO_2_**, and (**d**) **2PyNO_2_Cl** BODIPYs. Orbital energies in eV.

**Figure A2:**
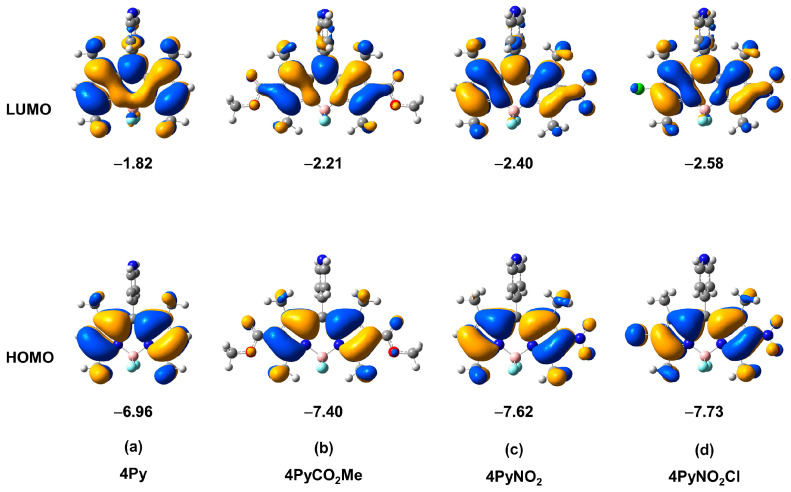
Frontier orbitals of (**a**) **4Py [29]**, (**b**) **4PyCO_2_Me**, (**c**) **4PyNO_2_**, and (**d**) **4PyNO_2_Cl** BODIPYs. Orbital energies in eV.

The similarity in the observed absorption and emission wavelengths correlates with the similarity in the calculated absorption and emission wavelengths and the almost identical HOMO–LUMO gaps observed within a given series (see Table 2). For the entire series of 8(*meso*)-pyridyl-BODIPYs, the HOMO is almost entirely localized on the BODIPY core, while the LUMO partially involves the pyridyl group (Figure 2, Figure A1 and Figure A2). This is consistent with our previous findings for the symmetric 2,6-unsubstituted and 2,6-dichloro-substituted 8(*meso*)-pyridyl-BODIPYs [29]. The shapes of the LUMO orbitals reflect the electron-withdrawing effect of the methyl ester and nitro substituents with the nitro effect being much more significant.

The absorption and emission bands for **2PyCO_2_Me**, **3PyCO_2_Me**, and **4PyCO_2_Me** appear at approximately 501 and 514 nm, respectively, in polar solvents, similar to those observed for the 2,6-unsubstituted 8(*meso*)-pyridyl-BODIPYs [29]. In the case of the mono-nitro BODIPYs **2PyNO_2_**, **3PyNO_2_**, and **4PyNO_2_**, the absorption and emission bands are blue-shifted, appearing at approximately 491 and 510 nm, respectively, in polar solvents. This result was confirmed with computational modeling (see Table 2) and is due to the slightly larger HOMO–LUMO gap induced by the strong electron-withdrawing nitro substituent. The introduction of a chlorine group on the mono-nitro BODIPYs causes significant red-shifted absorption and emission bands by ca. 20 nm and reduces the molar absorptivity of the absorption bands, as previously observed [29]. The absorption and emission bands of **2PyNO_2_Cl**, **3PyNO_2_Cl**, and **4PyNO_2_Cl** appear at approximately 511 and 527 nm in polar solvents. This is consistent with the smaller HOMO–LUMO gaps for these compounds compared to the mono-nitro BODIPYs.

The Stokes shifts observed in the polar solvents, methanol, and acetonitrile are similar, indicating similar changes in geometry following excitations within a given 2,6-substituted series. The mono-nitro BODIPYs display the largest Stokes shifts of all the studied compounds. These observed shifts agree with the performed computational studies (see Table 2) and are likely due to greater geometry changes in the case of the nitro-BODIPYs excited states. These effects are currently under investigation in our laboratory.

As previously observed in the 2,6-unsubstituted 8(*meso*)-pyridyl-BODIPYs [29], their fluorescence properties largely depend on the relative position of the nitrogen atom on the pyridine ring. The 2-pyridyl BODIPY derivatives consistently show the lowest fluorescence quantum yields due to the lower rotational barrier of the 2-pyridyl group and the closer proximity of the nitrogen atom to the BODIPY core in these compounds. On the other hand, the 3-pyridyl BODIPY derivatives display the largest fluorescence quantum yields closely followed by the 4-pyridyl BODIPY derivatives, as previously observed [29].

The presence of two methyl ester substituents at the 2,6-positions of the 8(*meso*)-pyridyl-BODIPYs increased their relative fluorescence quantum yields in acetonitrile and methanol. This is due to the ester groups decreasing the electron density on the BODIPY core. However, the introduction of a single nitro substituent on the BODIPY core significantly decreases the relative fluorescence quantum yields for all 8(*meso*)-pyridyl-BODIPYs, particularly in polar solvents, probably due to non-radiative deactivation pathways that are more pronounced in polar media. The presence of a single strongly electron-withdrawing nitro group dramatically increases the calculated dipole moment of the molecule, as shown in Table 2. On the other hand, when a chlorine atom is introduced into the mono-nitro BODIPYs, the fluorescence is partially restored due to the reduction in the polarity of the molecule.

## 3. Materials and Methods

### 3.1. Synthesis and Characterization

#### 3.1.1. General

Commercially available reagents and solvents were used as received from VWR or Sigma Aldrich unless noted otherwise. All reactions were monitored with thin-layer chromatography (TLC) using 0.2 mm silica gel plates (with UV indicator, polyester backed, 60 Å, pre-coated). Liquid chromatography was performed on preparative TLC plates or via silica gel column chromatography (60 Å, 230–400 mesh). NMR spectra were measured on 400 or 500 MHz for ^1^H, 400 MHz for ^11^B NMR, and 500 MHz for ^13^C spectrometer. Chemical shifts (δ) are given in parts per million (ppm) in CDCl_3_ (7.27 ppm for ^1^H NMR, 77.0ppm for ^13^C NMR) or (CD_3_)_2_CO (2.05 ppm for ^1^H NMR, 206.68 and 29.92 ppm for ^13^C NMR) or CD_2_Cl_2_ (5.32 ppm for ^1^H NMR, 53.5 ppm for ^13^C NMR); coupling constants (J) are given in hertz. BF_3_·OEt_2_ was used as the reference (0.00 ppm) for ^11^B NMR spectra. High-resolution mass spectra (HRMS) were obtained using an Agilent 6230-B ESI-TOF mass spectrometer.

**2,4-Dimethyl-3-methoxycarbonyl-pyrrole** [32] and BODIPYs **2Py** [18], **3Py** [27], and **4Py** [33] were prepared as previously reported, and their spectroscopic data agree with the literature reports.

#### 3.1.2. 1,3,5,7-Tetramethyl-2,6-dimethoxycarbonyl-8-(2-pyridyl)-BODIPY (**2PyCO_2_Me**)

To a solution of 2,4-dimethyl-3-methyl carbonyl pyrrole in dichloromethane (0.984 g, 6.42 mmol) in a 250 mL round-bottomed flask under nitrogen was added 2-pyridyl carboxaldehyde (0.31 mL, 3.21 mmol) and 2–5 drops of TFA. The reaction mixture was stirred at rt for 40 h. DDQ (0.729 g, 3.21 mmol) was added to the reaction mixture. After 2 h, Et_3_N (4.48 mL, 32.12 mmol) was added to the reaction mixture followed by BF_3_·OEt_2_ (3.96 mL, 32.12 mmol). The reaction was stirred for another 48 h. The reaction mixture was washed with water, and NaHCO_3_ and the organic layers were extracted with dichloromethane. The combined organic layers were then washed with brine, dried over Na_2_SO_4_, and the solvent evaporated under reduced pressure. Purification with column chromatography using 20–40% ethyl acetate/hexanes for elution gave 0.567 g, 45% of the titled BODIPY as a reddish solid. ^1^H NMR (500 MHz, CDCl_3_) δ 8.85 (d, *J* = 4.8 Hz, 1H, Ar–H), 7.92 (t, *J* = 7.7 Hz, 1H, Ar–H), 7.57–7.49 (m, 1H, Ar–H), 7.46 (d, *J* = 7.7 Hz, 1H, Ar–H), 3.83 (s, 6H, CO_2_CH_3_), 2.85 (s, 6H, CH_3_), 1.60 (s, 6H, CH_3_). ^13^C NMR (126 MHz, CDCl_3_) δ 164.6, 160.4, 153.1, 150.5, 147.4, 137.6, 131.5, 124.6, 124.4, 51.4, 15.3, 13.2, 13.1. ^11^B NMR (128 MHz, CD_2_Cl_2_) δ 0.72 (t, *J* = 32.0 Hz). HRMS (ESI) *m*/*z* calcd (%) for C_22_H_22_BF_2_N_3_O_4_: 441.1783 [M + H]^+^; found 442.1746.

#### 3.1.3. 1,3,5,7-Tetramethyl-2,6-dimethoxycarbonyl-8-(3-pyridyl)-BODIPY (**3PyCO_2_Me**)

This BODIPY was prepared as described above for **2PyCO_2_Me** using 2,4-dimethyl-3-methoxycarbonylpyrrole (0.307 g, 2.00 mmol), 3-pyridinecarboxaldehyde (0.1 mL, 1.00 mmol), TFA (5 drops), DDQ (0.454 g, 2.00 mmol), Et_3_N (2.09 mL, 15.01 mmol), and BF_3_·OEt_2_ (1.85 mL, 15.01 mmol). Purification with column chromatography using dichloromethane/ethyl acetate/hexanes 3:1:6 afforded the title BODIPY 0.353 g, 40% yield. ^1^H NMR (500 MHz, CDCl_3_) δ 8.89–8.83 (m, 1H, Ar–H), 8.60 (s, 1H, Ar–H), 7.72 (d, *J* = 7.8 Hz, 1H, Ar–H), 7.63–7.57 (m, 1H, Ar–H), 3.82 (s, 6H, CO_2_CH_3_), 2.84 (s, 6H, CH_3_), 1.65 (s, 6H, CH_3_). ^13^C NMR (126 MHz, CDCl_3_) δ 164.4, 160.6, 147.3, 137.6, 131.5, 124.9, 123.1, 122.9, 51.6, 15.2, 15.1, 14.5, 14.4. ^11^B NMR (128 MHz, CDCl_3_) δ 0.66 (t, *J* = 31.7 Hz). HRMS (ESI) *m*/*z* calcd (%) for C_22_H_22_BF_2_N_3_O_4_: 441.1783 [M + H]^+^; found 442.1771.

#### 3.1.4. 1,3,5,7-Tetramethyl-2,6-dimethoxycarbonyl-8-(4-pyridyl)-BODIPY (**4PyCO_2_Me**)

This BODIPY was prepared as described above for **2PyCO_2_Me** using 2,4-dimethyl-3-methoxycarbonylpyrrole (1.630 g, 10.64 mmol), 4-pyridinecarboxaldehyde (0.5 mL, 5.32 mmol), TFA (5 drops), DDQ (1.2078 g, 5.32 mmol), triethylamine (11.12 mL, 79.81 mmol), and BF_3_·OEt_2_ (9.85 mL, 79.81 mmol). Purification with column chromatography using 60% ethyl acetate/hexanes 3:1:6 afforded the title BODIPY in 0.232 g, 28% yield. ^1^H NMR (500 MHz, CDCl_3_) δ 8.90 (d, 4.8Hz, 2H, Ar–H), 7.48 (d, *J* = 5.0 Hz, 2H, Ar–H), 3.82 (s, 6H, CO_2_CH_3_), 2.84 (s, 6H, CH_3_), 1.67 (s, 6H, CH_3_). ^13^C NMR (126 MHz, CD_2_Cl_2_) δ 164.7, 160.5, 151.5, 147.7, 143.1, 142.4, 130.9, 123.4, 123.3, 51.2, 14.8, 13.9, 13.6, 13.5. ^11^B NMR (128 MHz, CD_2_Cl_2_) δ 0.65 (t, *J* = 32.1 Hz). HRMS (ESI) *m*/*z* calcd (%) for C_22_H_22_BF_2_N_3_O_4_: 441.1783 [M + H]^+^; found 442.1743.

#### 3.1.5. 2-Nitro-1,3,5,7-tetramethyl-8-(2-pyridyl)-BODIPY (**2PyNO_2_**)

1,3,5,7-Tetramethyl-8-(2-pyridyl)-BODIPY (20 mg, 0.0615 mmol) was dissolved in 10 mL of dry dichloromethane in an oven-dried 50 mL round-bottomed flask. Nitronium tetrafluoroborate (0.12 mL, 0.06 mmol) was then added, and the mixture was stirred for 5 h at rt. The solvent was evaporated under reduced pressure, and the crude product was dissolved in methyl tert-butyl methyl ether and then washed with water. The organic layer was dried over Na_2_SO_4_, and the solvent was evaporated under reduced pressure. The reaction was purified via preparative TLC using 40% ethyl acetate/hexanes, yielding 18.72 mg, 82% yield of the title BODIPY as a reddish solid. ^1^H NMR (500 MHz, acetone-*d*_6_) δ 8.85 (dt, *J* = 4.9, 1.4 Hz, 1H, Ar–H), 8.09 (dt, *J* = 7.7, 1.7 Hz, 1H, Ar–H), 7.76 (dt, *J* = 7.8, 1.2 Hz, 1H, Ar–H), 7.66 (ddd, *J* = 7.7, 4.9, 1.2 Hz, 1H, Ar–H), 6.49 (s, 1H,pyrrolic-H), 2.80 (s, 3H, CH_3_), 2.65 (s, 3H, CH_3_), 1.56 (s, 3H, CH_3_), 1.42 (s, 3H, CH_3_). ^13^C NMR (126 MHz, acetone-*d*_6_) δ 153.2, 151.5, 149.9, 142.2, 138.7, 126.4, 126.3, 126.3, 126.3, 125.9, 125.6, 15.4, 14.5, 14.3, 11.9. ^11^B NMR (128 MHz, acetone-*d*_6_) δ 0.57 (t, *J* = 31.5 Hz). HRMS (ESI) *m*/*z* calcd (%) for C_18_H_17_BF_2_N_4_O_2_: 371.1489 [M + H]^+^; found 371.1494.

#### 3.1.6. 2-Nitro-1,3,5,7-tetramethyl-8-(3-pyridyl)-BODIPY (**3PyNO_2_**)

This BODIPY was prepared as described above for **2PyNO_2_** using 1,3,5,7-tetramethyl-8-(3-pyridyl)-BODIPY [25] (40mg, 0.12 mmol) and NO_2_BF_4_ (0.25 mL, 0.12 mmol). The residue was purified via preparative TLC with ethyl acetate/hexanes 1:1, yielding 37.44 mg, 82% yield of the title BODIPY as a reddish solid. ^1^H NMR (500 MHz, acetone-*d*_6_) δ 8.88 (dd, *J* = 4.9, 1.7 Hz, 1H, Ar–H), 8.75 (d, *J* = 2.2 Hz, 1H, Ar–H), 8.02 (dt, *J* = 7.8, 2.0 Hz, 1H, Ar–H), 7.71 (dd, *J* = 7.8, 4.9 Hz, 1H, Ar–H), 6.54 (s, 1H, pyrrolic-H), 2.80 (s, 3H, CH_3_), 2.66 (s, 3H, CH_3_), 1.68 (s, 3H, CH_3_), 1.50 (s, 3H, CH_3_). ^13^C NMR (126 MHz, acetone-*d*_6_) δ 151.9, 149.1, 137.1, 130.9, 126.7, 126.7, 125.1, 15.8, 15.4, 14.3, 12.9. ^11^B NMR (128 MHz, acetone-*d*_6_) δ 0.53 (t, *J* = 31.5 Hz). HRMS (ESI) *m*/*z* calcd (%) for C_18_H_17_BF_2_N_4_O_2_: 371.1489 [M + H]^+^; found 371.1490.

#### 3.1.7. 2-Nitro-1,3,5,7-tetramethyl-8-(4-pyridyl)-BODIPY (**4PyNO_2_**)

This compound was prepared as described above for **2PyNO_2_** using 1,3,5,7-tetramethyl-8-(4-pyridyl)-BODIPY (0.030 g, 0.09 mmol) and NO_2_BF_4_ (0.18 mL, 0.09 mmol). Purification with preparative TLC using dichloromethane/ethyl acetate/hexanes 3:2:5 solvent afforded 0.023 g, 77% yield of the title BODIPY as a reddish solid. ^1^H NMR (500 MHz, acetone-*d*_6_) δ 8.90 (d, *J* = 5.0 Hz, 2H, Ar–H), 7.65 (d, *J* = 4.9 Hz, 2H, Ar–H), 6.54 (s, 1H, pyrrolic-H), 2.80 (s, 3H, CH_3_), 2.66 (s, 3H, CH_3_), 1.71 (s, 3H, CH_3_), 1.55 (s, 3H, CH_3_). ^13^C NMR (126 MHz, CDCl_3_) δ 166.8, 147.6, 147.3, 137.5, 134.1, 133.9, 126.4, 125.1, 22.8, 15.6, 12.9, 11.2. ^11^B NMR (128 MHz, acetone-*d*_6_) δ 0.49 (t, *J* = 31.3 Hz). HRMS (ESI) *m*/*z* calcd (%) for C_18_H_17_BF_2_N_4_O_2_: 371.1489 [M + H]^+^; found 371.1492.

#### 3.1.8. 2-Chloro-6-nitro-1,3,5,7-tetramethyl-8-(2-pyridyl)-BODIPY (**2PyNO_2_Cl**)

BODIPY **2PyNO_2_** (11 mg, 0.0297 mmol) was dissolved in dry degassed dichloromethane in a 10 mL oven-dried round-bottomed flask. TCCA (0.003 g, 0.01 mmol) in dry dichloromethane was added dropwise to the solution. The mixture was stirred at room temperature for 2 h and purified with preparative TLC using 40% ethyl acetate/hexanes for elution to afford 0.010 g, 90% yield of the title BODIPY as a pink reddish product. ^1^H NMR (500 MHz, CDCl_3_) δ 8.88 (d, *J* = 4.9 Hz, 1H, Ar–H), 8.05–7.99 (m, 1H, Ar–H), 7.65–7.61 (m, 1H, Ar–H), 7.51 (t, *J* = 5.8 Hz, 1H, Ar–H), 2.88 (s, 3H, CH_3_), 2.69 (s, 3H, CH_3_), 1.60 (s, 3H, CH_3_), 1.36 (s, 3H, CH_3_). ^13^C NMR (126 MHz, CDCl_3_) δ 160.9, 152.4, 151.4, 150.8, 141.4, 141.2, 137.8, 136.2, 132.9, 131.1, 128.9, 127.4, 125.1, 124.5, 14.7, 14.3, 12.2, 12.1. ^11^B NMR (128 MHz, acetone-*d*_6_) δ 0.37 (t, *J* = 30.9 Hz). HRMS (ESI) *m*/*z* calcd (%) for C_18_H_16_BClF_2_N_4_O_2_: 403.106 [M + H]^+^; found 404.1130.

#### 3.1.9. 2-Chloro-6-nitro-1,3,5,7-tetramethyl-8-(3-pyridyl)-BODIPY (**3PyNO_2_Cl**)

This BODIPY was prepared as described above for **2PyNO_2_Cl** using **3PyNO_2_** (12.7 mg, 0.0343 mmol) and TCCA (0.004 g, 0.02 mmol). The reaction was stirred for 30 min and purified with preparative TLC using dichloromethane/ethyl acetate/hexanes 3:2:5 for elution to afford 0.013 g, 89% yield of the title BODIPY as a pink reddish product. ^1^H NMR (500 MHz, acetone-*d*_6_) δ 8.89 (dd, *J* = 4.9, 1.7 Hz, 1H, Ar–H), 8.77 (d, *J* = 2.3 Hz, 1H, Ar–H), 8.04 (dt, *J* = 7.8, 2.0 Hz, 1H,Ar–H), 7.75–7.70 (m, 1H, Ar–H), 2.80 (s, 3H, CH_3_), 2.67 (s, 3H, CH_3_), 1.68 (s, 3H, CH_3_), 1.47 (s, 3H, CH_3_). ^13^C NMR (126 MHz, acetone-*d*_6_) δ 159.9, 151.2, 148.1, 142.1, 141.7, 136.2, 135.9, 132.9, 129.8, 124.3, 13.5, 12.4, 12.3, 12.2. ^11^B NMR (128 MHz, acetone-*d*_6_) δ 0.34 (t, *J* = 30.8 Hz). HRMS (ESI) *m*/*z* calcd (%) for C_18_H_16_BClF_2_N_4_O_2_: 403.106 [M + H]^+^; found 404.1134.

#### 3.1.10. 2-Chloro-6-nitro-1,3,5,7-tetramethyl-8-(4-pyridyl)-BODIPY (**4PyNO_2_Cl**)

This BODIPY was prepared as described above for **2PyNO_2_Cl** using **4PyNO_2_** (15 mg, 0.0405 mmol) and TCCA (0.003 g, 0.01 mmol). The reaction was stirred for 2 h. Column chromatography was performed using dichloromethane/ethyl acetate/hexanes 3:2:5 for elution to afford 0.013 g, 89% yield of the title BODIPY as a pink reddish product. ^1^H NMR (400 MHz, CDCl_3_) δ 8.90 (d, *J* = 6.1 Hz, 2H, Ar–H), 7.39 (d, *J* = 6.0 Hz, 2H, Ar–H), 2.88 (s, 3H, CH_3_), 2.70 (s, 3H, CH_3_), 1.72 (s, 3H, CH_3_), 1.46 (s, 3H, CH_3_). ^13^C NMR (126 MHz, CDCl_3_) δ 161.8, 151.9, 148.7, 140.5, 139.1, 135.9, 131.7, 126.1, 124.3, 14.6, 14.3, 13.5, 13.1. ^11^B NMR (128 MHz, acetone-*d*_6_) δ 0.37 (t, *J* = 30.9 Hz). HRMS (ESI) *m*/*z* calcd (%) for C_18_H_16_BClF_2_N_4_O_2_: 403.106 [M + H]^+^; found 404.1134.

### 3.2. Spectroscopy Methods

UV–vis absorption spectra were collected on a Varian Cary 50 Bio spectrophotometer. Emission spectra were obtained on a PerkinElmer LS55 spectrophotometer at room temperature. Spectrophotometric grade solvents and quartz cuvettes (1 cm path length) were used. Relative fluorescence quantum yields (Φ_f_) were calculated using rhodamine 6G (Φ_f_ = 0.86 in methanol) as the reference using the following equation: Φ_x_ = Φ_st_ × Grad_x_/Grad_st_ × (η _x_/η _st_)^2^, where Φ_X_ and Φ_ST_ are the quantum yields of the sample and standard, Grad_X_ and Grad_ST_ are the gradients from the plot of integrated fluorescence intensity vs. absorbance, and η represents the refractive index of the solvent (x is for the sample and st standard).

### 3.3. X-ray Crystallography

The structures were determined using data collected at low temperature on a Bruker Kappa ApexII DUO diffractometer with CuKα radiation for **2PyCO_2_Me** (90 K), **2PyNO_2_** (100 K), **4PyNO_2_** (100 K), **2PyNO_2_Cl** (100 K), **3PyNO_2_Cl** (100 K), and **4PyNO_2_Cl** (100 K), or with MoKα radiation for **4PyCO_2_Me** (120 K) and **3PyNO_2_** (100 K). Disorder was present in several of the structures, and disordered solvent contribution was removed using the SQUEEZE procedure for **3PyNO_2_**. Data in CIF format have been deposited with the Cambridge Crystallographic Data Centre as CCDC 2249642-2249649 in the order shown in Figure 1.

### 3.4. Computational Methods

The geometries of the ground states of all compounds were optimized without symmetry constraints at the -b3lyp/6-31+G(d,p) level in dichloromethane. The solvent effects were considered using the Polarized Continuum Model (PCM). The potential energy minima were confirmed with frequency calculations. The absorption and emission data were calculated using the TD-DFT/6-31+G(d,p) method in vacuum. This method has been shown to correctly reproduce the experimental trends [28]. The first three singlet excitations were considered, and the lowest-energy excited singlet state was optimized to calculate the maximum emission wavelengths. All calculations were performed using the Gaussian 09 program package [34].

## 4. Conclusions

Three series of 8(*meso*)-pyridyl-BODIPYs bearing a 2-, 3-, or-4-pyridyl group and electron-withdrawing groups at either the 2- or 2,6-positions were synthesized, and their structural and spectroscopic properties were investigated. These BODIPYs were prepared either with direct electrophilic nitration or chlorination of the pyridyl-BODIPY core or by total synthesis from *tert*-butyl acetoacetate. Eight of the new BODIPYs were characterized with X-ray crystallography, and their structures were modeled computationally. All 8(*meso*)-pyridyl rings are nearly perpendicular to the BODIPY core with dihedral angles between 80° and 90°, slightly lower in the case of the 2- versus the 3- and 4-pyridyl derivatives.

The 2,6-methyl ester groups in **2PyCO_2_Me**, **3PyCO_2_Me**, and **4PyCO_2_Me** were observed to increase the relative fluorescence quantum yields of these derivatives compared with the corresponding 2,6-unsubstituted analogs due to the electron-withdrawing effect of the methyl ester groups. On the other hand, the introduction of a 2-nitro substituent on the 8(*meso*)-pyridyl-BODIPYs drastically increases the calculated dipole moment of the molecules, induces significant hypsochromic shifts, and decreases their relative fluorescence quantum yields due to non-radiative deactivation processes. Introduction of a chlorine atom in the series **2PyNO_2_C**l, **3PyNO_2_Cl**, and **4PyNO_2_Cl** reduces the polarity of the molecules relative to the mono-nitro compounds, induces pronounced bathochromic shifts, and partially restores the fluorescence.

## Figures and Tables

**Table 1:** Spectroscopic properties of *meso*-pyridyl-BODIPYs in CH_3_CN and CH_3_OH.

Solvent	BODIPY	λ_abs_ (nm)	λ_em_ (nm)	Stokes Shift (nm)	Φ_f_^a^	ε (M^−1^ cm^−1^)
CH_3_CN	**2Py** ^b^	502	514	12	0.04	87,500
	**3Py** ^b^	502	514	12	0.43	92,900
	**4Py** ^c^	501	515	14	0.31	72,100
	**2PyCO_2_Me**	501	515	14	0.09	64,160
	**3PyCO_2_Me**	501	512	11	0.61	87,690
	**4PyCO_2_Me**	500	510	10	0.43	84,500
	**2PyNO_2_**	491	509	18	0.08	55,810
	**3PyNO_2_**	491	507	16	0.26	54,740
	**4PyNO_2_**	490	508	18	0.25	43,450
	**2PyNO_2_/Cl**	512	527	15	0.13	23,790
	**3PyNO_2_/Cl**	512	526	14	0.36	23,850
	**4PyNO_2_/Cl**	511	525	14	0.28	16,510
CH_3_OH	**2PyCO_2_Me**	502	516	14	0.21	88,200
	**3PyCO_2_Me**	501	514	13	0.61	70,760
	**4PyCO_2_Me**	501	516	15	0.39	72,880
	**2PyNO_2_**	491	511	20	0.09	64,750
	**3PyNO_2_**	491	510	19	0.13	587,240
	**4PyNO_2_**	491	510	19	0.12	52,160
	**2PyNO_2_/Cl**	511	530	19	0.20	37,730
	**3PyNO_2_/Cl**	511	529	18	0.35	51,110
	**4PyNO_2_/Cl**	511	529	18	0.32	41,050

^a^ Calculated using rhodamine 6G (Φ_f_ = 0.86) in methanol at λ_exc_ = 473 nm as the standard. ^b^ Previous work from this laboratory [29]. ^c^ Previous work from this laboratory [28].

**Table 2:** CAM-B3LYP/6-31+G(d,p) calculated spectroscopic and electronic properties of pyridyl BODIPYs (in vacuum). All transitions are S_0_-S_1_.

BODIPY	λ_abs_ (nm)	Oscillator Strength	HOMO (eV)	LUMO (eV)	HOMO-LUMO Gap (eV)	Dipole Moment (D)	λ_em_ (nm)	Stokes Shift (nm)
**2Py** ^a^	416	0.542	−6.77	−1.62	5.14	6.05	430	14
**3Py** ^a^	416	0.542	−6.92	−1.78	5.14	3.94	429	13
**4Py** ^a^	415	0.543	−6.96	−1.82	5.15	2.13	428	12
**2PyCO_2_Me**	413	0.797	−7.21	−2.02	5.19	3.00	436	23
**3PyCO_2_Me**	413	0.793	−7.36	−2.18	5.18	1.89	430	17
**4PyCO_2_Me**	412	0.794	−7.40	−2.21	5.19	1.05	434	22
**2PyNO_2_**	408	0.655	−7.44	−2.22	5.21	9.51	436	28
**3PyNO_2_**	407	0.656	−7.58	−2.37	5.21	8.02	429	22
**4PyNO_2_**	406	0.660	−7.62	−2.40	5.22	7.54	431	25
**2PyNO_2_Cl**	417	0.673	−7.55	−2.40	5.14	8.14	443	26
**3PyNO_2_Cl**	416	0.675	−7.69	−2.55	5.14	6.44	438	21
**4PyNO_2_Cl**	416	0.676	−7.73	−2.58	5.15	5.66	440	24

^a^ Previous work from this laboratory [29].

## Data Availability

Not applicable.

## Supplementary Materials

The following supporting information can be downloaded at: https://www.mdpi.com/article/10.3390/molecules28124581/s1, Figure S1, conformations and relative energies of BODIPYs studied; Figures S2 and S3, normalized absorption (a,c,e) and emission (b,d,f) spectra of BODIPYs in acetonitrile (a, b, c, d) and water (e, f); Table S1, spectroscopic properties determined in toluene using rhodamine-6G (Φ = 0.86), λ_exc_ = 473 nm; Figures S4–S30, ^1^H, ^13^C, and ^11^B NMR spectra of BODIPYs.
