# Supplementary material for: 8(meso)-Pyridyl-BODIPYs: Effects of 2,6-Substitution with Electron-Withdrawing Nitro, Chloro, and Methoxycarbonyl Groups
Source: Molecules. 2023 Jun 6;28(12):4581. doi: 10.3390/molecules28124581 (PMC10303842; doi:10.3390/molecules28124581)
Supplement: Supplementary file 1 [file molecules-28-04581-s001.zip › molecules-2402249-supplementary.pdf]

Supplemental Materials

8(*meso*)-Pyridyl-BODIPYs: Effects of 2,6-substitution with Electron-Withdrawing Nitro, Chloro, and Methoxycarbonyl Groups

Caroline Ndung'U [1], Petia Bobadova-Parvanova [2], Daniel LaMaster [1], Dylan Goliber [2], Frank R. Fronczek [1] and Maria da Graça H. Vicente[1,*]

Table of Contents

Spectroscopic Properties 2

BODIPYs conformations and relative energies 2

Absorption Spectra 3

Emission Spectra 4

NMR spectra 5

Table S1. Spectroscopic properties of BODIPYs in Toluene and relative fluorescence quantum yields using (a) rhodamine 6G ($\Phi_f = 0.86$) in methanol, $\lambda_{exc} = 473$ nm

Solvent	BODIPY	λ_{abs} (nm)	λ_{em} (nm)	Stokes Shift (nm)	Φ_f	ε (M^{-1} cm^{-1})
Toluene	2PyCO$_2$Me	508	523	15	0.04	72285
	3PyCO$_2$Me	508	521	13	0.47	77490
	4PyCO$_2$Me	507	520	13	0.45	N/A
	2PyNO$_2$	501	518	17	0.03	27390
	3PyNO$_2$	500	517	17	0.5	53385
	4PyNO$_2$	500	517	17	0.38	57080
	2PyNO$_2$/Cl	520	538	18	0.05	30780
	3PyNO$_2$/Cl	521	536	15	0.47	22940
	4PyNO$_2$/Cl	520	537	17	0.42	20130

Figure S1. Conformations and relative energies for a) 2PyCO$_2$Me, b) 2PyNO$_2$, and c) 2PyNO$_2$Cl. Analogous conformations were also studied for the 3Py and 4Py series.

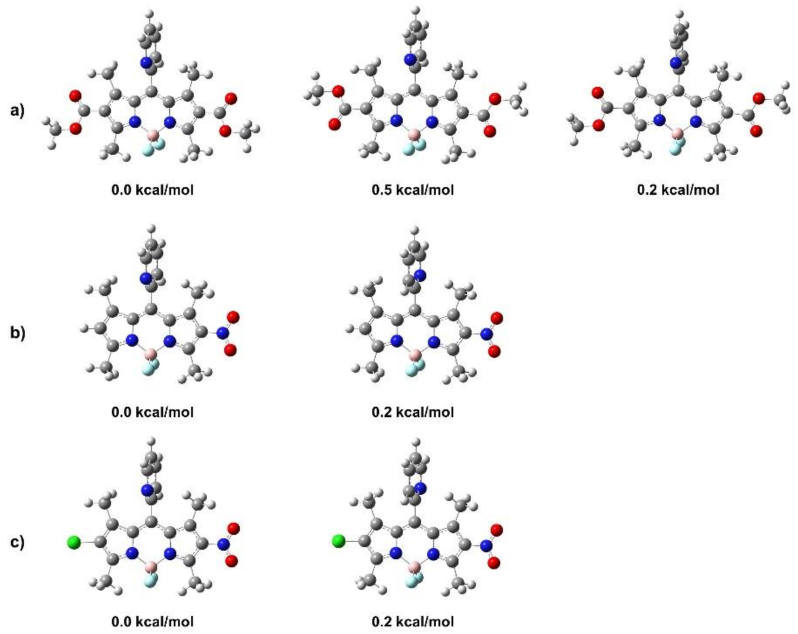

Figure S2. Normalized absorption spectra (1a, b) in CH_3CN (1c,1d) in MeOH and (1e,1f) in toluene.

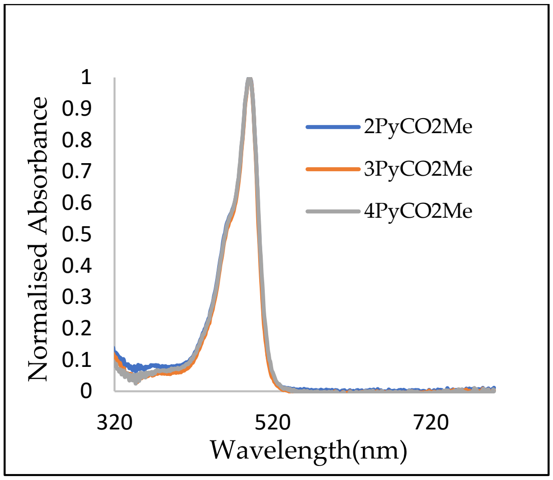

1a

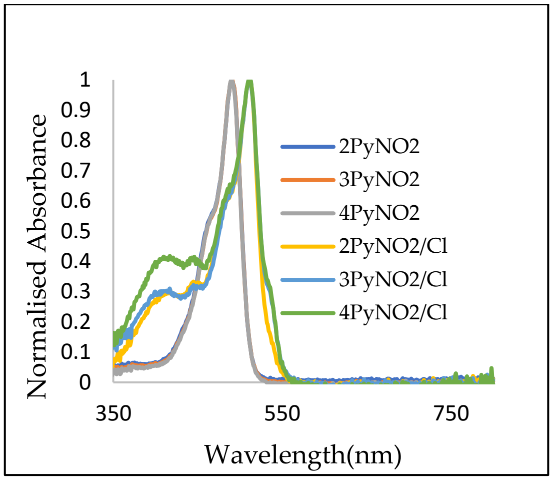

1b

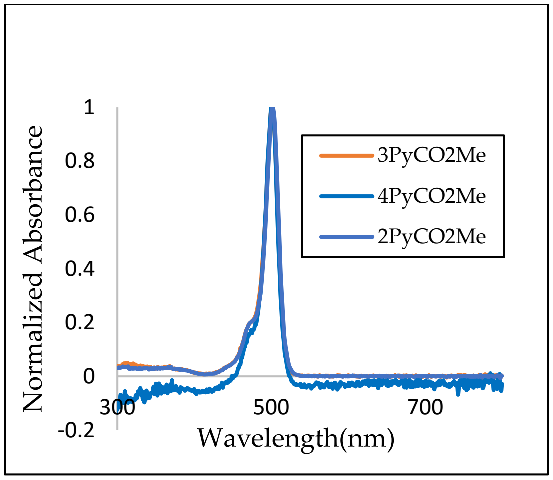

1c

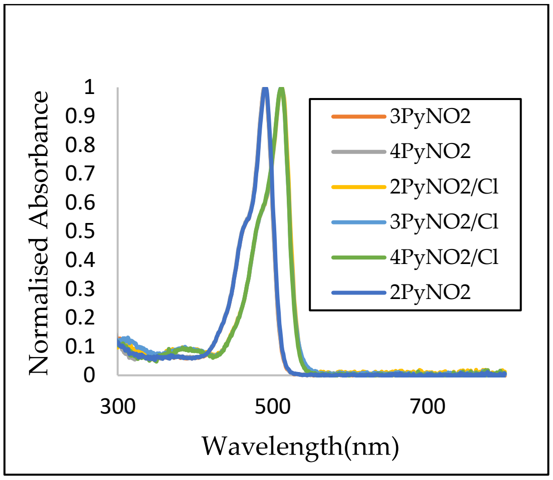

1d

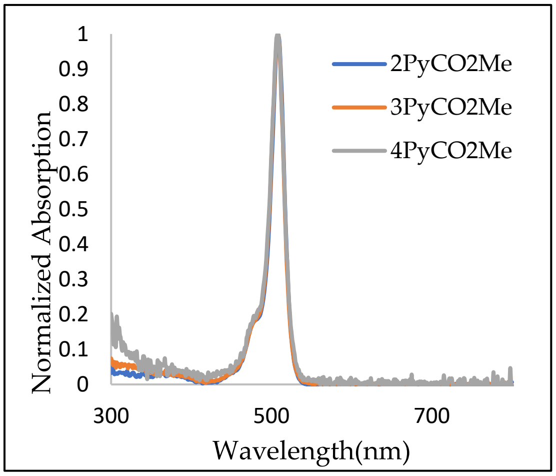

1e

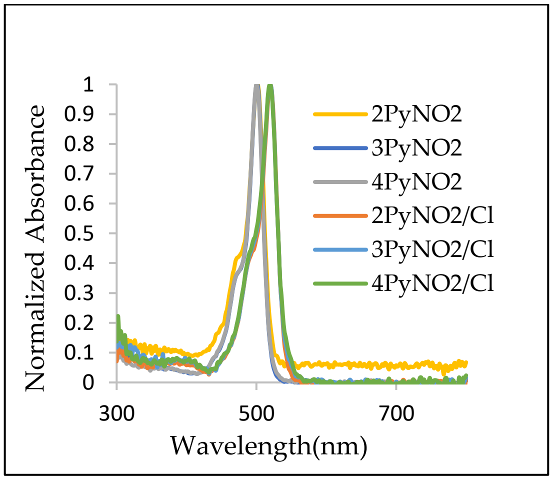

1f

Figure S3. Normalized emission spectra (2a, 2b) in CH_3CN (2c, 2d) in MeOH and (2e,2f) in toluene.

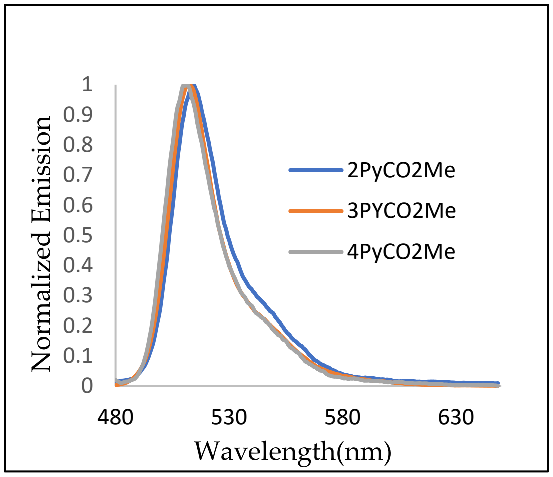

2a

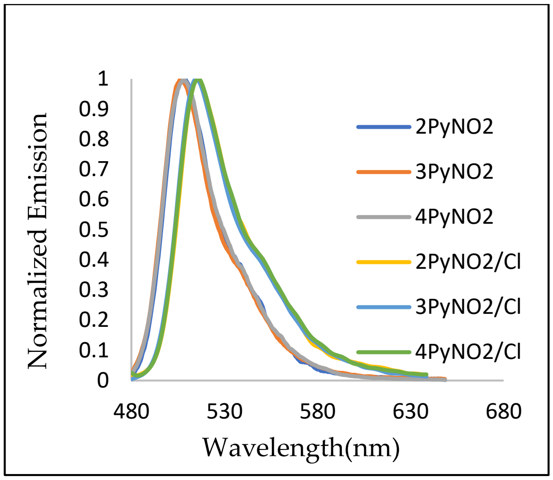

2b

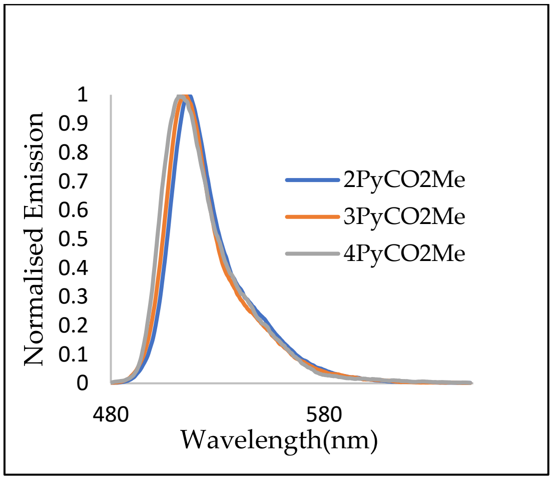

2c

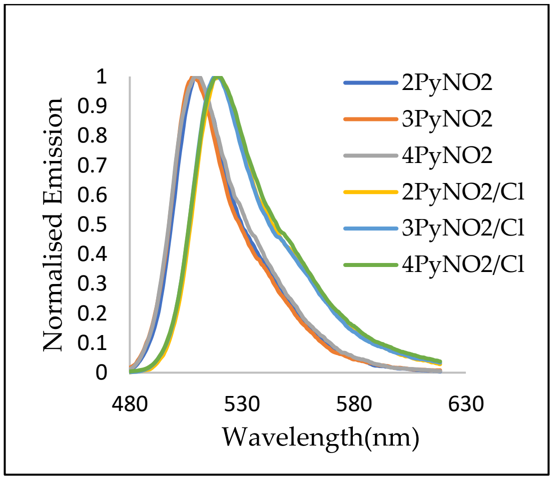

2d

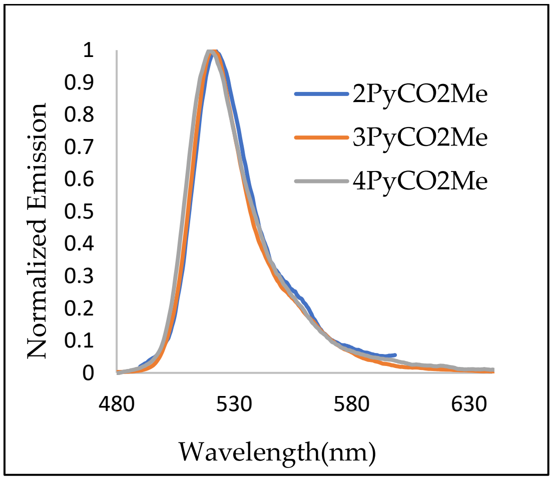

2e

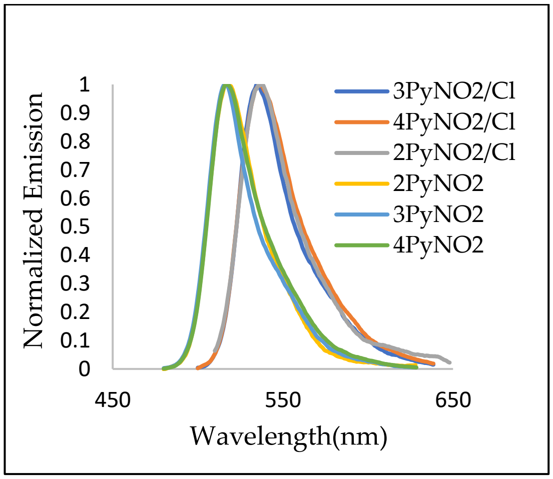

2f

NMR Spectra

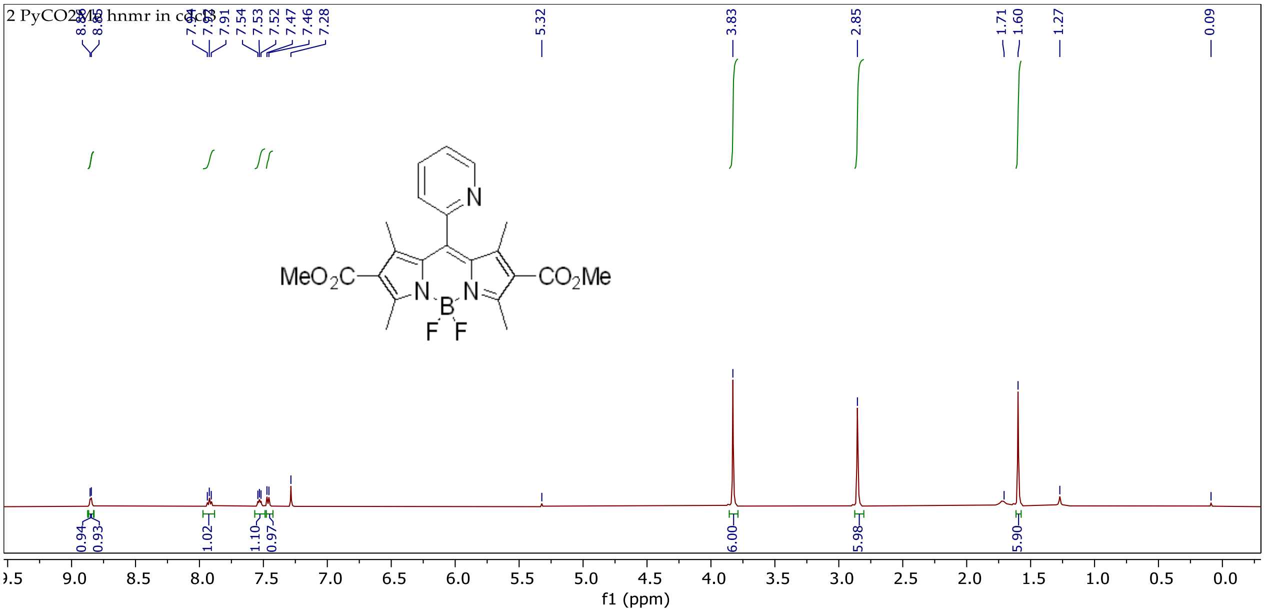

Figure S4. [1]H of BODIPY **2PyCO2Me** in **CDCl3**

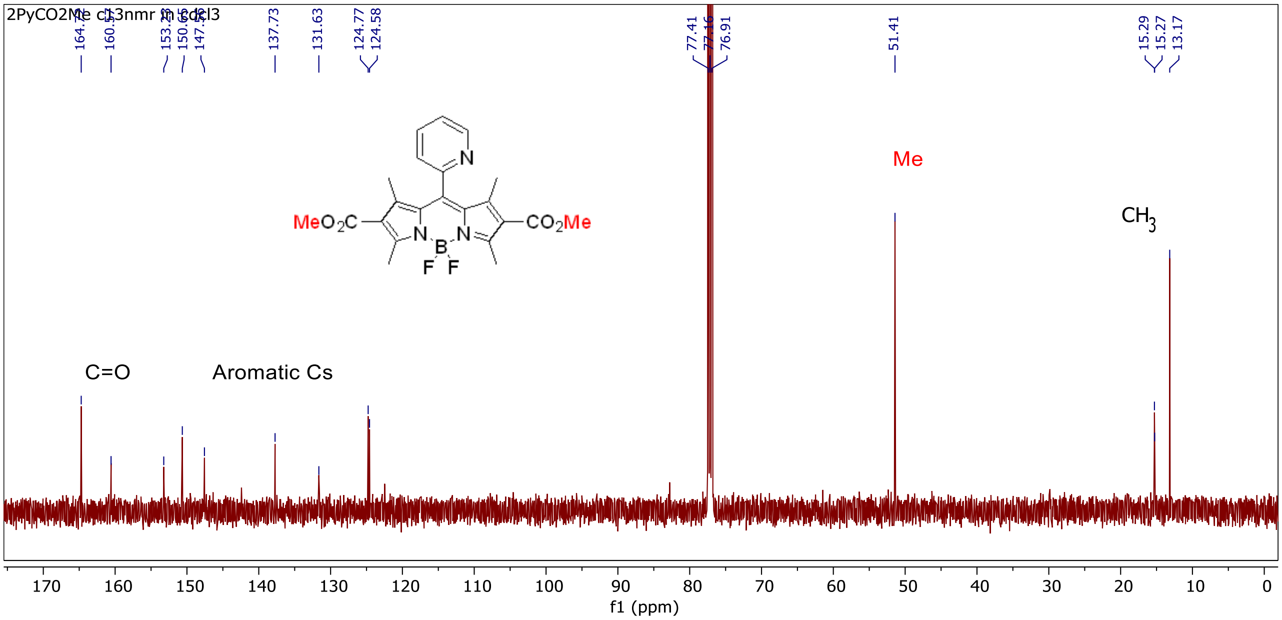

Figure S5. [13]C of BODIPY **2PyCO2Me** in **CDCl3**

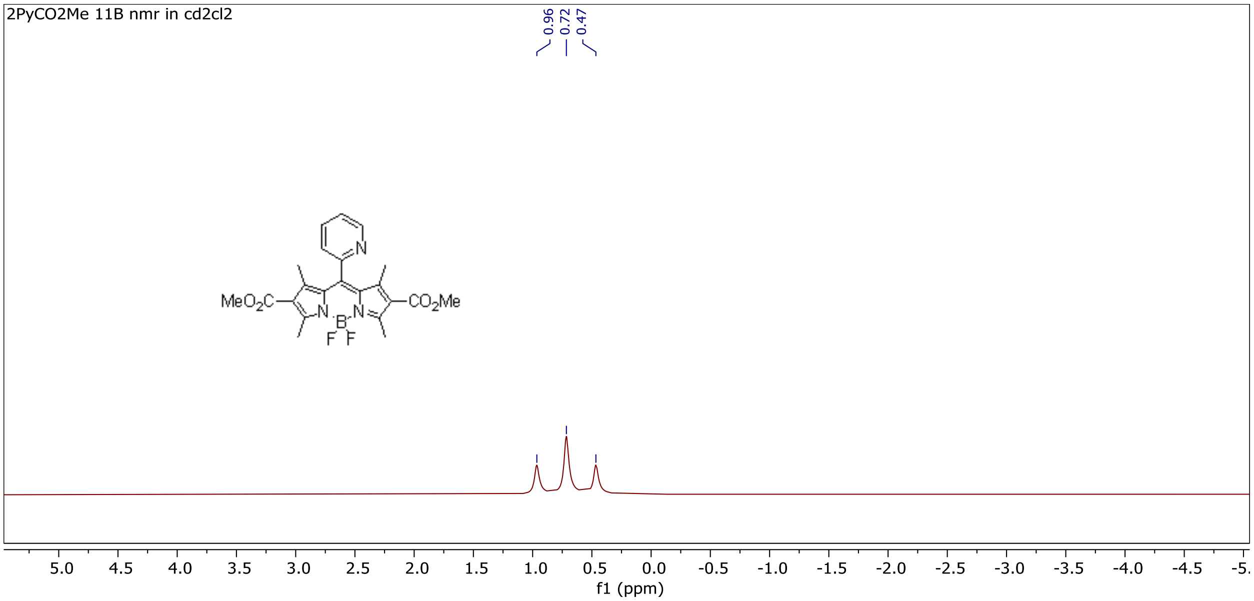

Figure S6. ^{11}B of BODIPY **$2PyCO_2Me$** in **CD_2Cl_2**

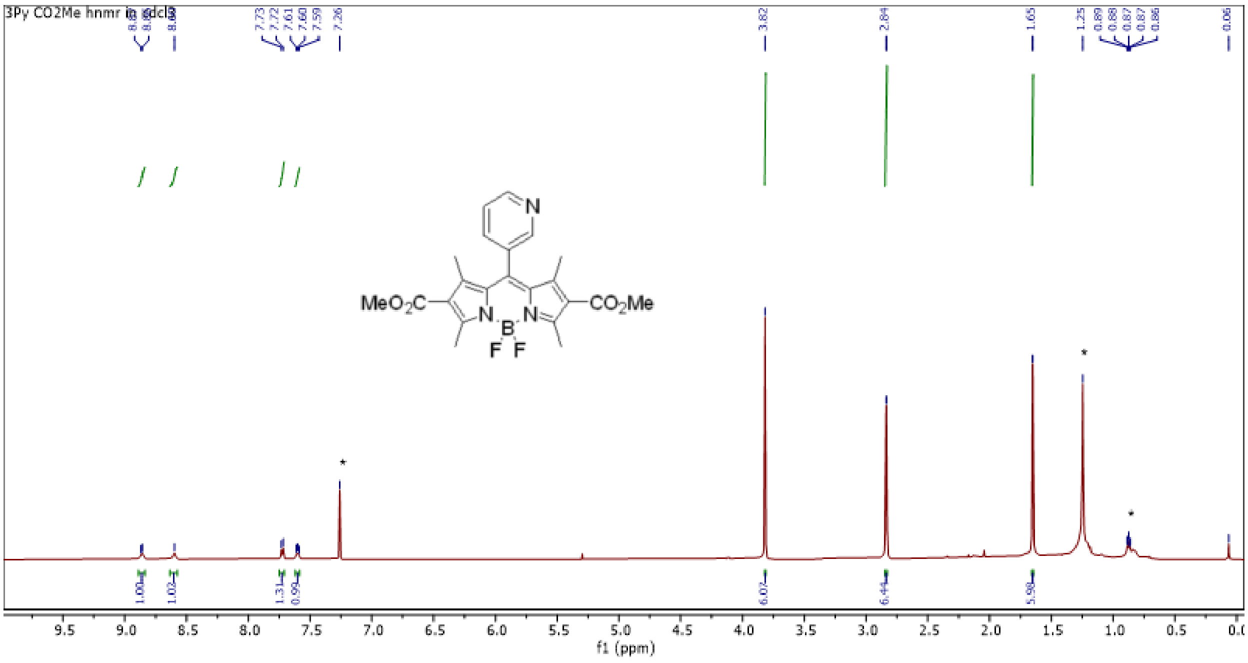

Figure S7. ^{1}H of BODIPY **$3PyCO_2Me$** in **$CDCl_3$**

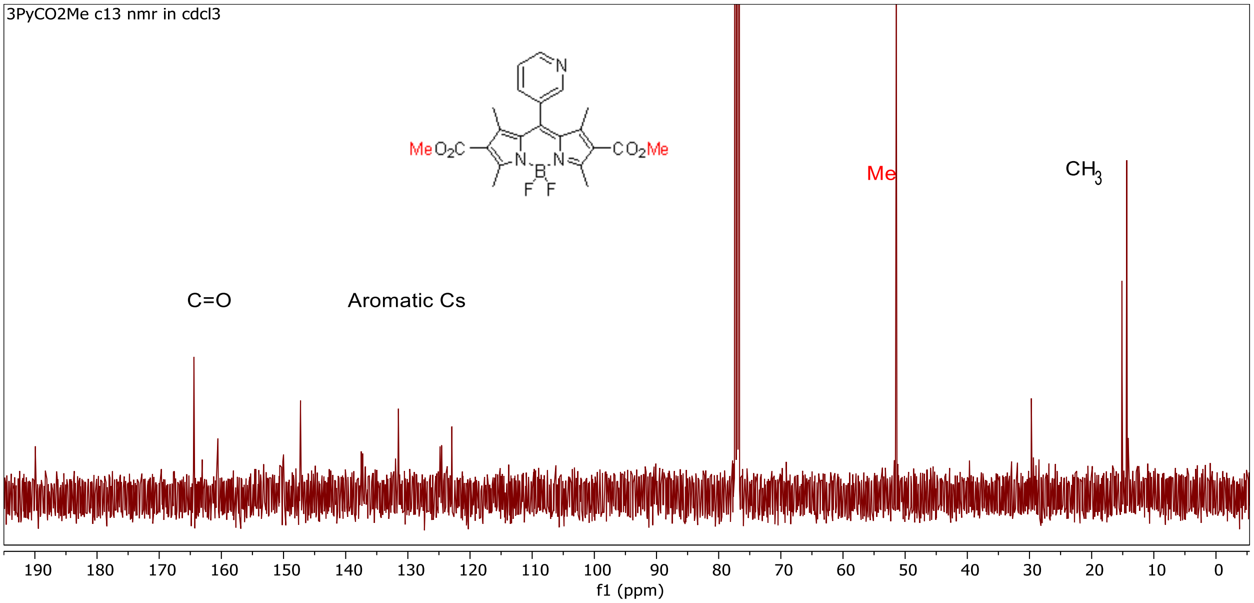

Figure S8. ^{13}C of BODIPY 3**PyCO$_2$Me** in **CDCl$_3$**

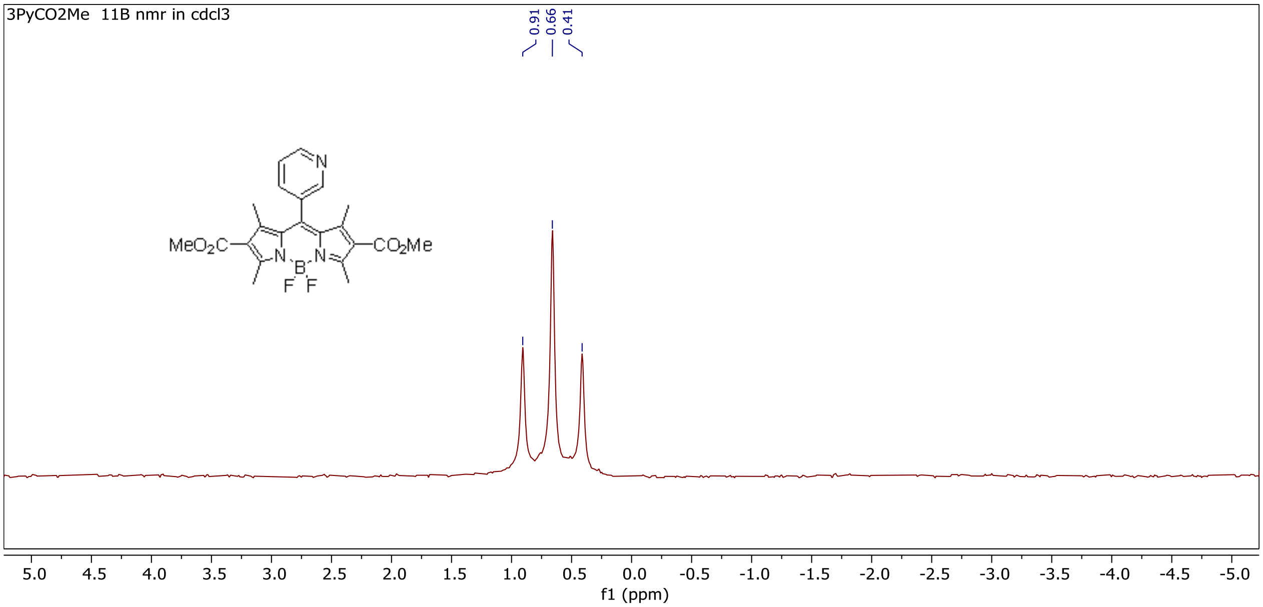

Figure S9. ^{11}B of BODIPY **3PyCO$_2$Me** in **CDCl$_3$**

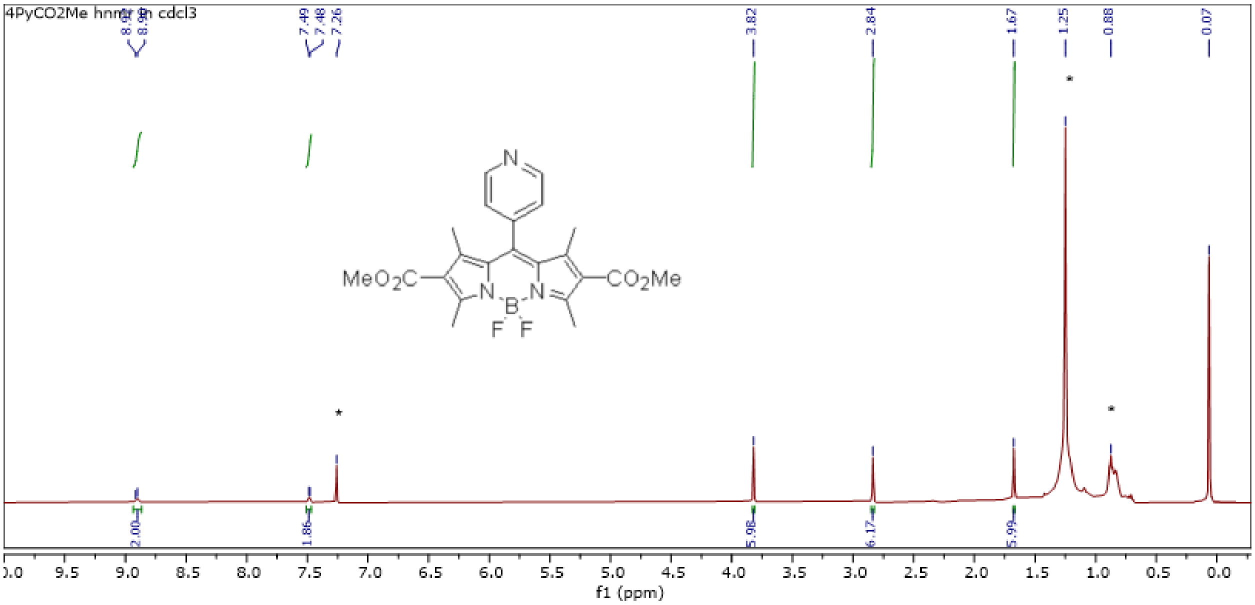

Figure S10. [1]H of BODIPY **4PyCO2Me** in **CDCl3**

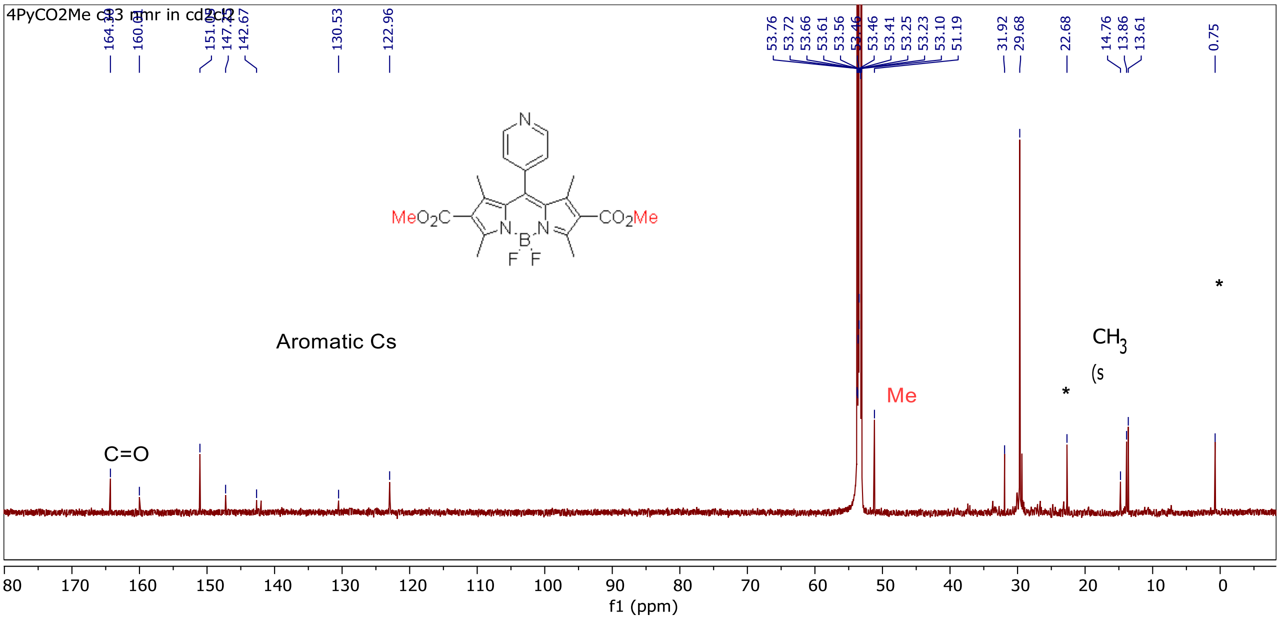

Figure S11. [13]C of BODIPY **4PyCO2Me** in **CD2Cl2**

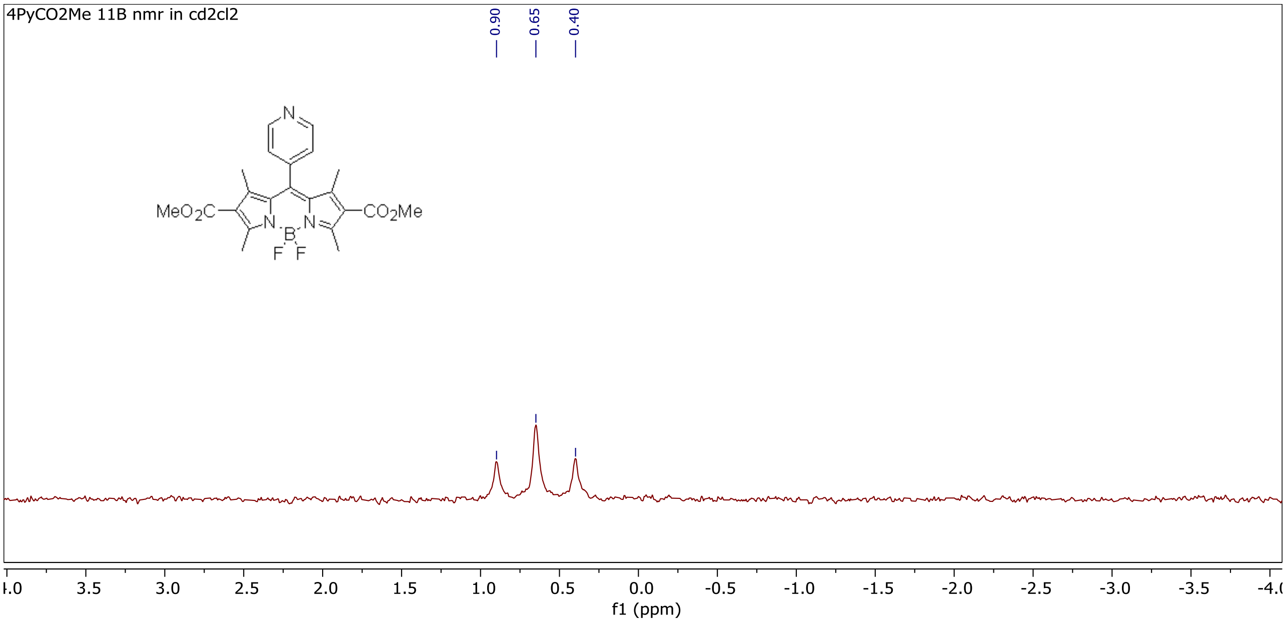

Figure S12. ^{11}B of BODIPY **4PyCO$_2$Me** in **CD$_2$Cl$_2$**

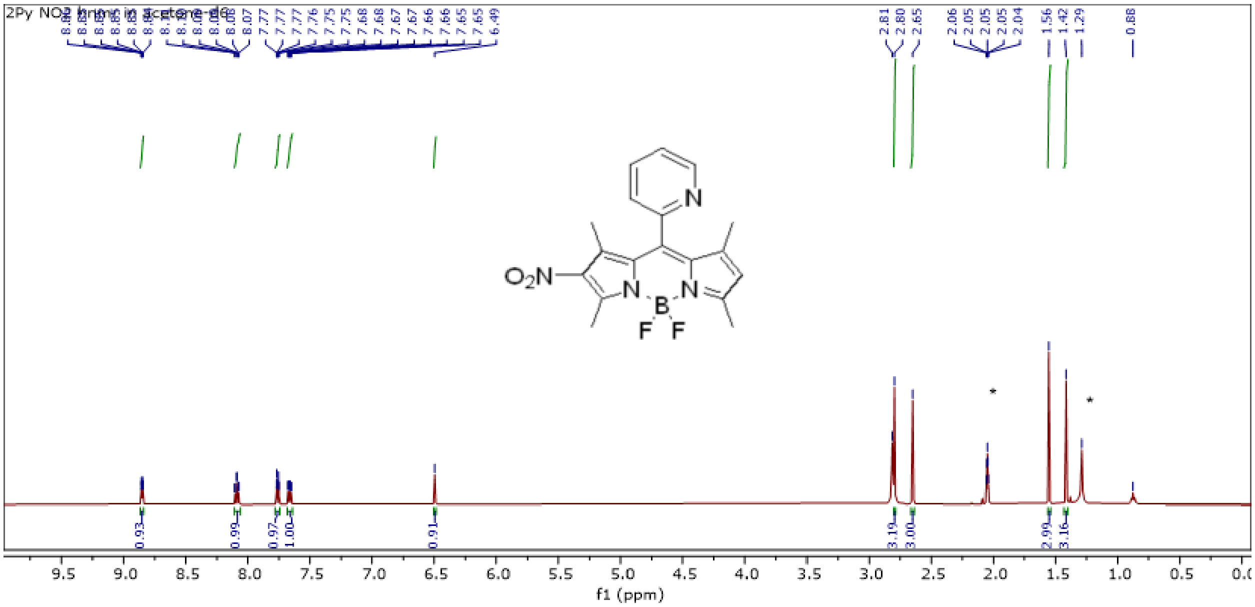

Figure S13. ^{1}H of BODIPY **2PyNO$_2$** in **acetone-d$_6$**

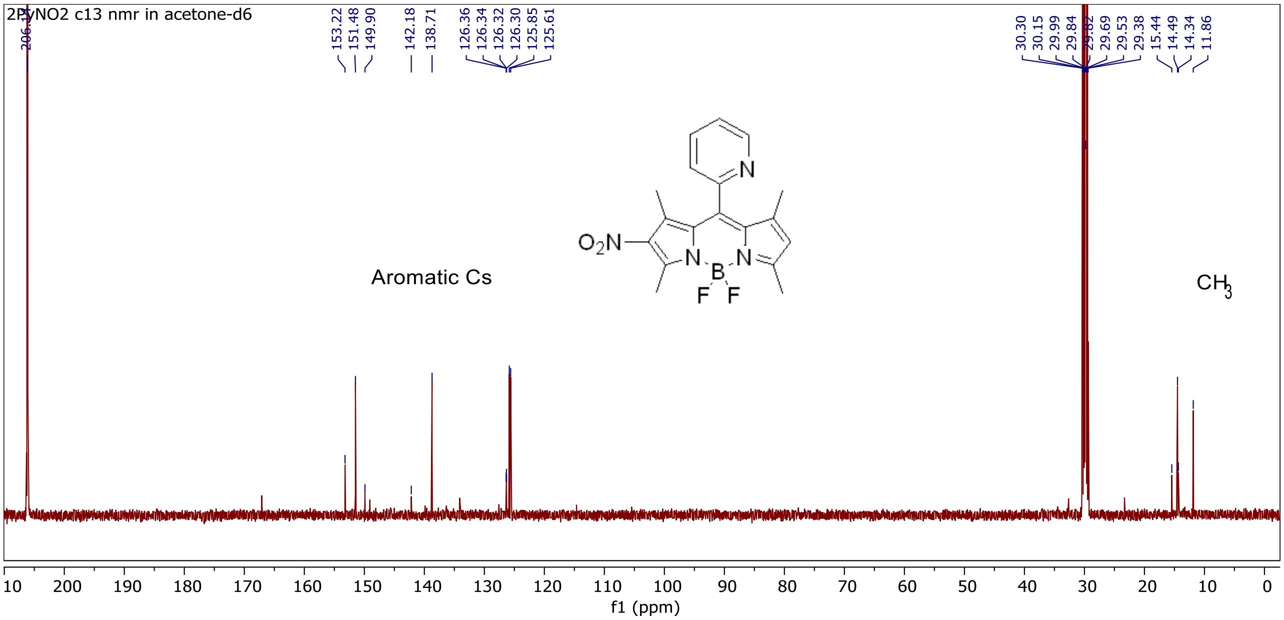

Figure S14. ^{13}C of BODIPY **2PyNO2** in **acetone-d6**

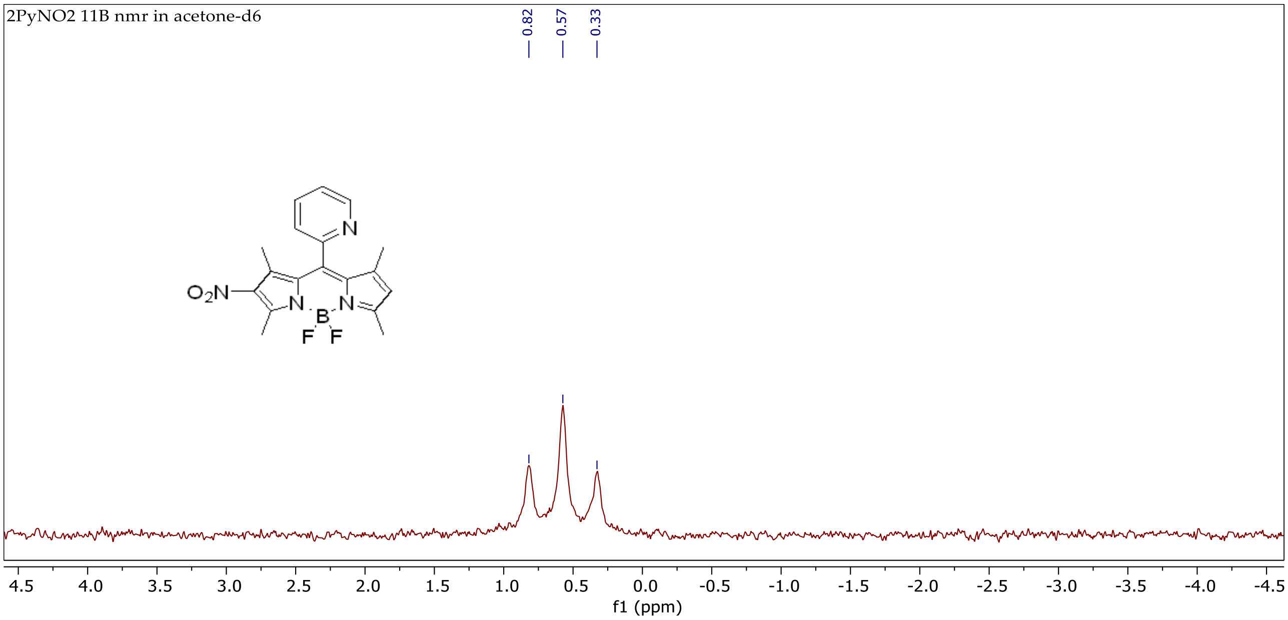

Figure S15. ^{11}B of BODIPY **2PyNO2** in **acetone-d6**

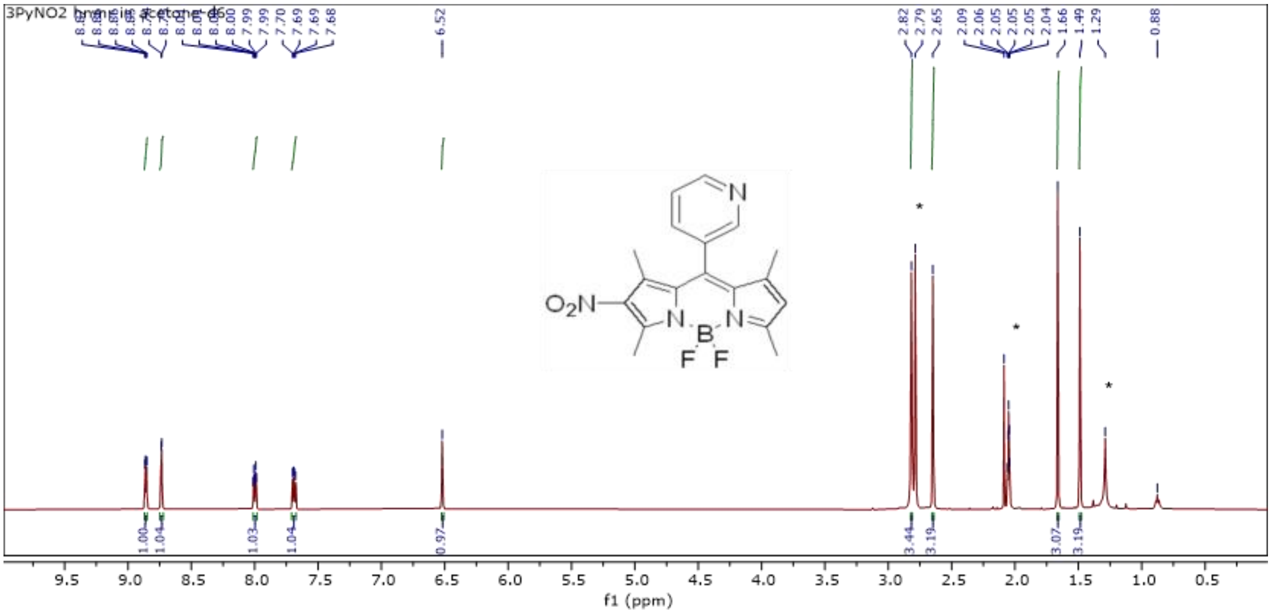

Figure S16. ^{1}H of BODIPY **3PyNO$_2$** in **acetone-d$_6$**

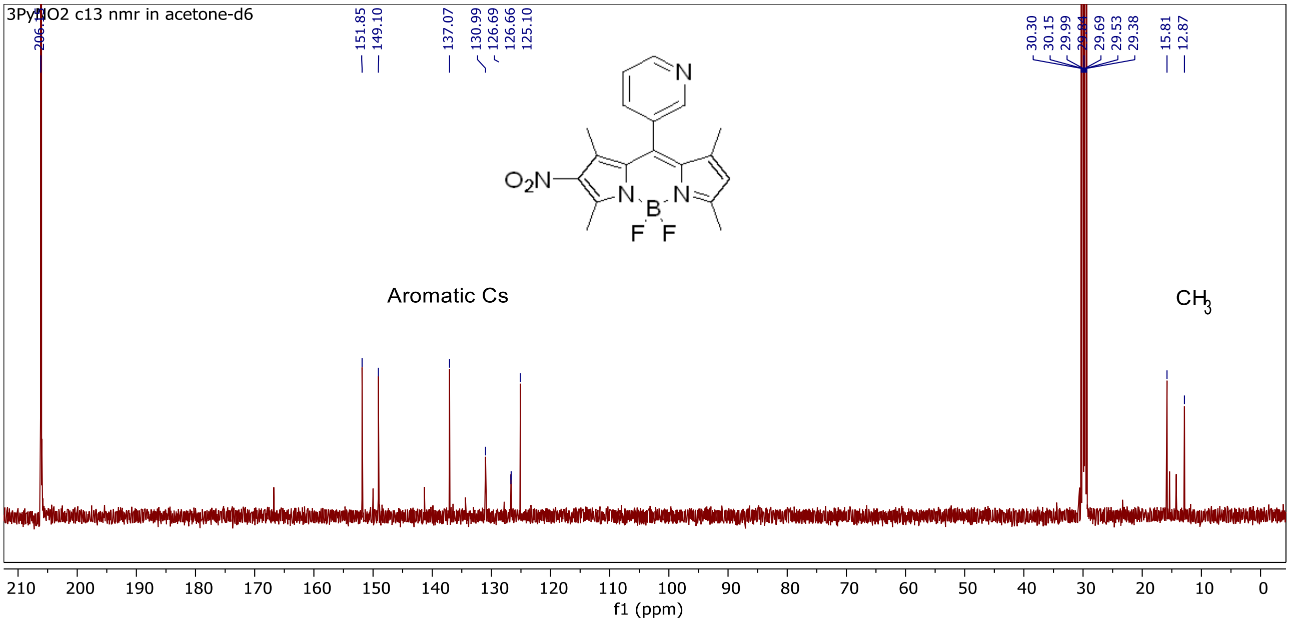

Figure S17. ^{13}C of BODIPY **3PyNO$_2$** in **acetone-d$_6$**

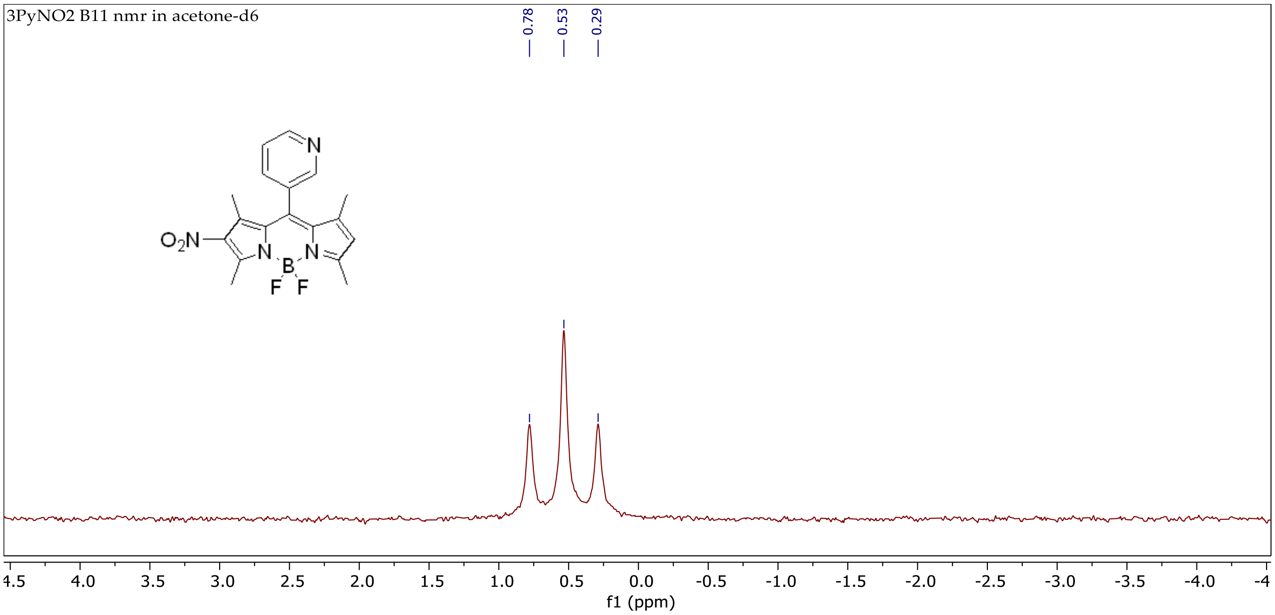

Figure S18. [11]B of BODIPY **3PyNO$_2$** in **acetone-d$_6$**

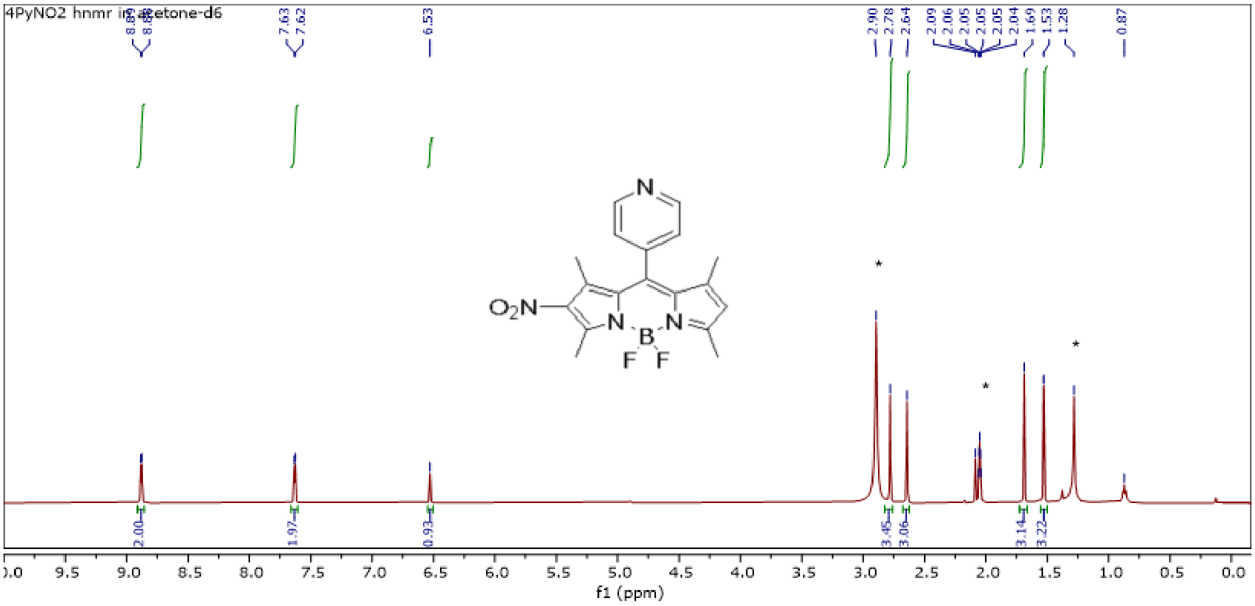

Figure S19. [1]H of BODIPY **4PyNO$_2$** in **Acetone-d6**

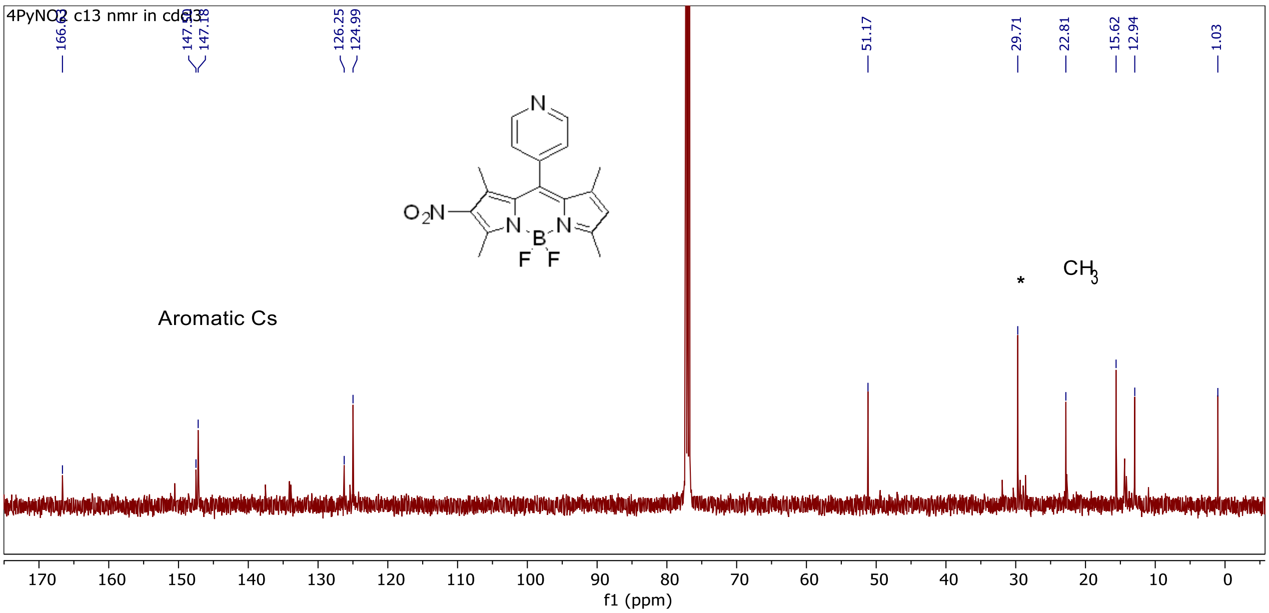

Figure S20. ^{13}C of BODIPY **4PyNO$_2$** in **CDCl$_3$**

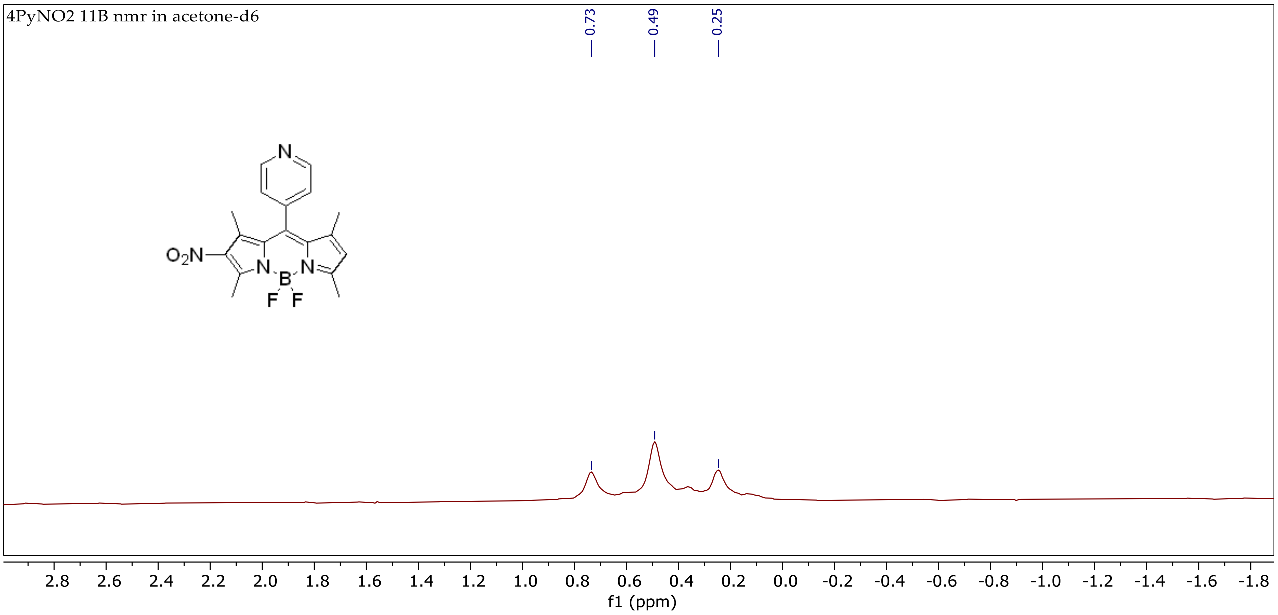

Figure S21. ^{11}B of BODIPY **4PyNO$_2$** in **acetone-d$_6$**

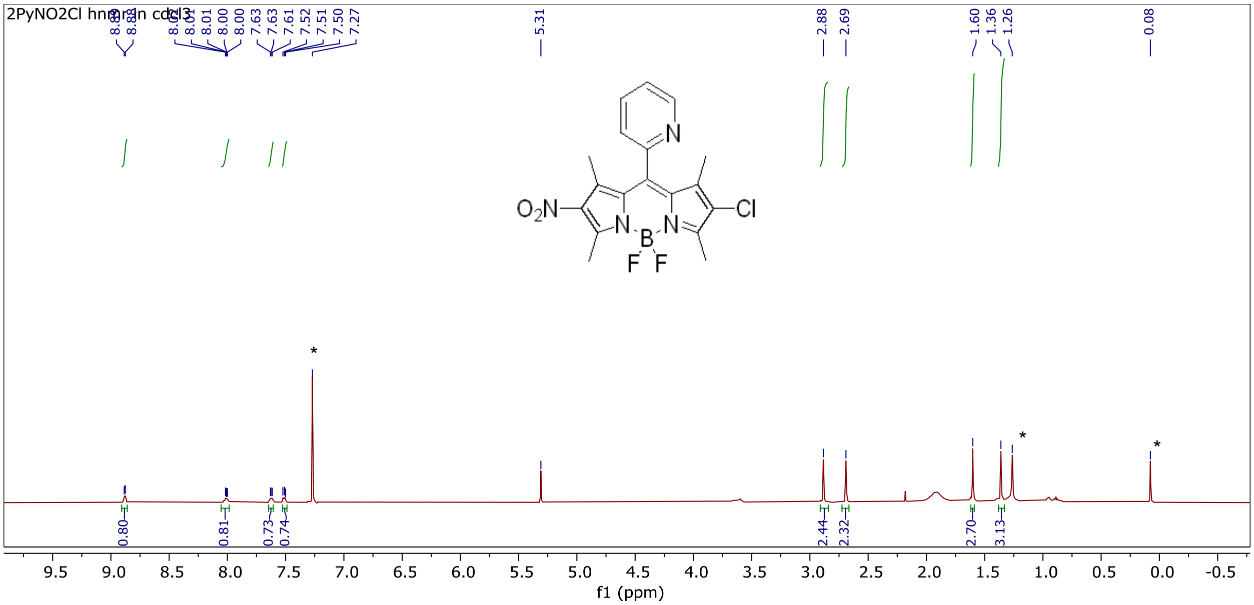

Figure S22. ^{1}H of BODIPY **2PyNO₂Cl** in **CDCl₃**

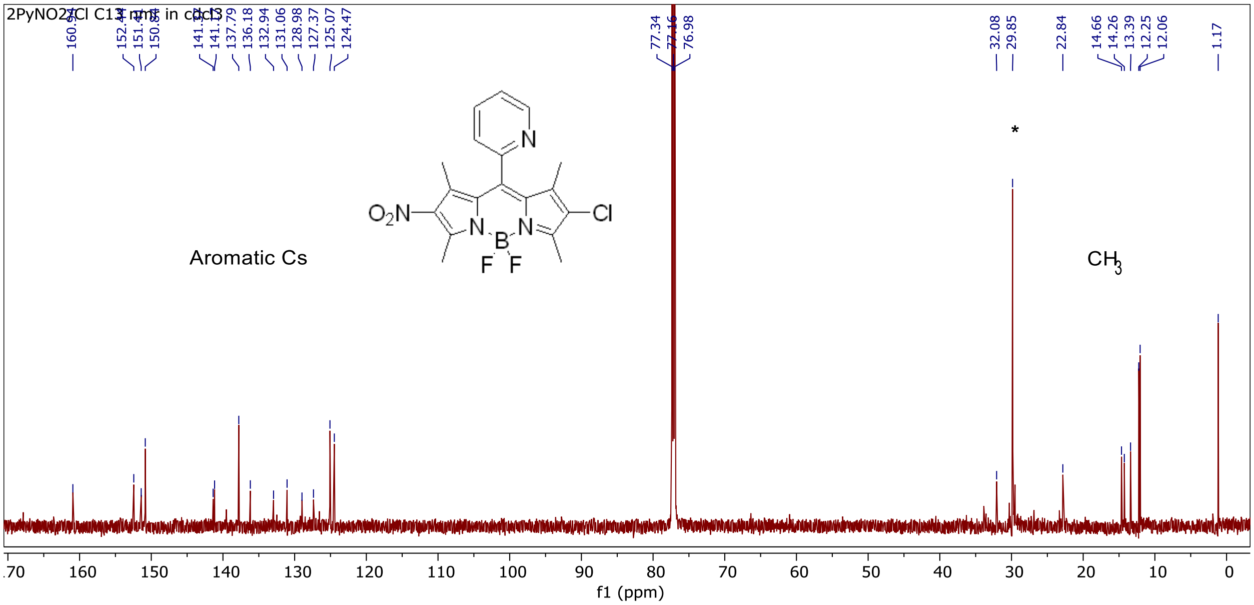

Figure S23. ^{13}C of BODIPY **2PyNO₂Cl** in **CDCl₃**

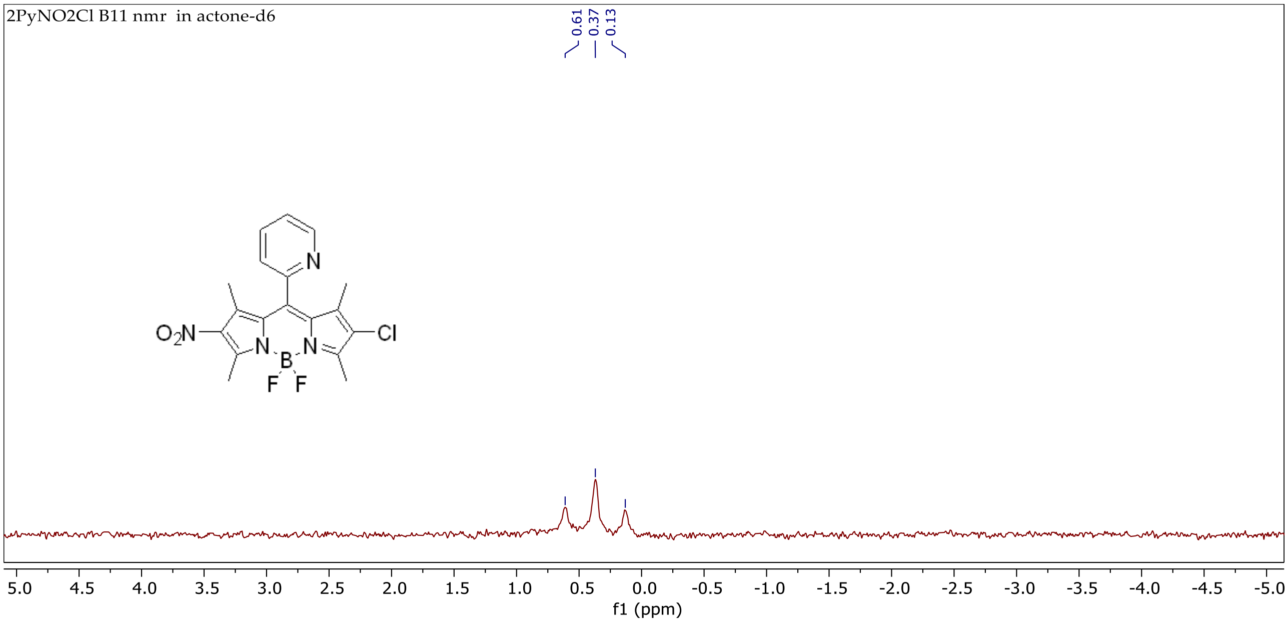

Figure S24. [11]B of BODIPY **2PyNO$_2$Cl** in **Acetone-d6**

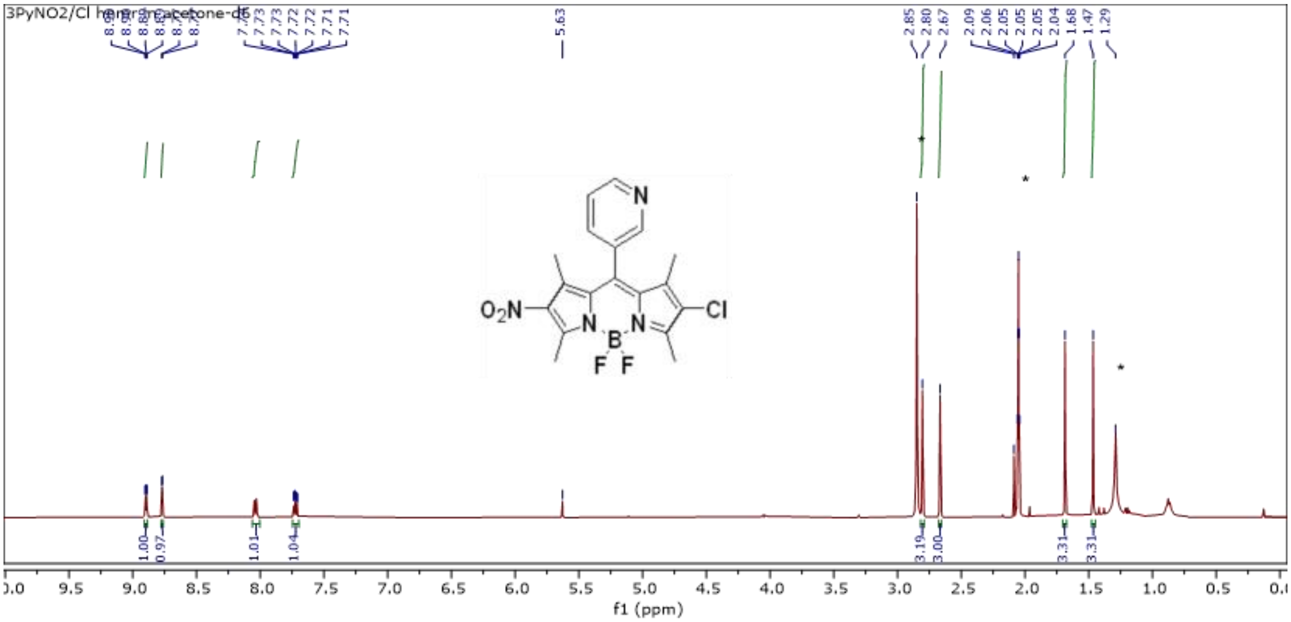

Figure S25. [1]H of BODIPY **3PyNO$_2$Cl** in **acetone-d$_6$**

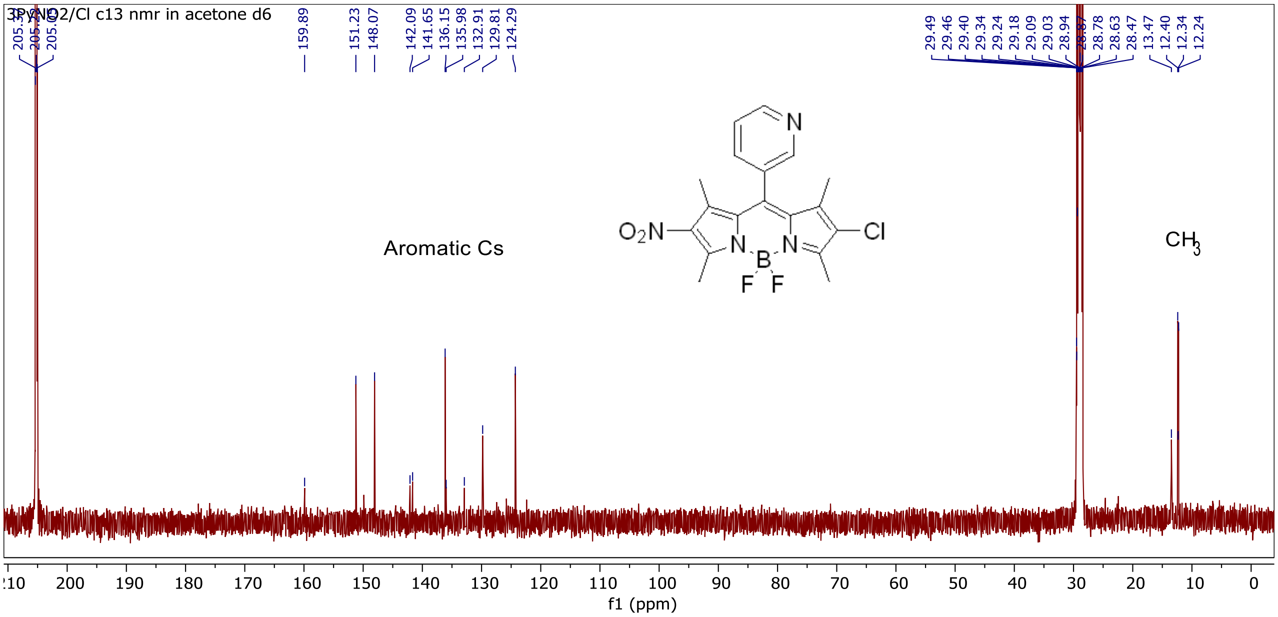

Figure S26. ^{13}C of BODIPY **3PyNO$_2$Cl** in **Acetone-d6**

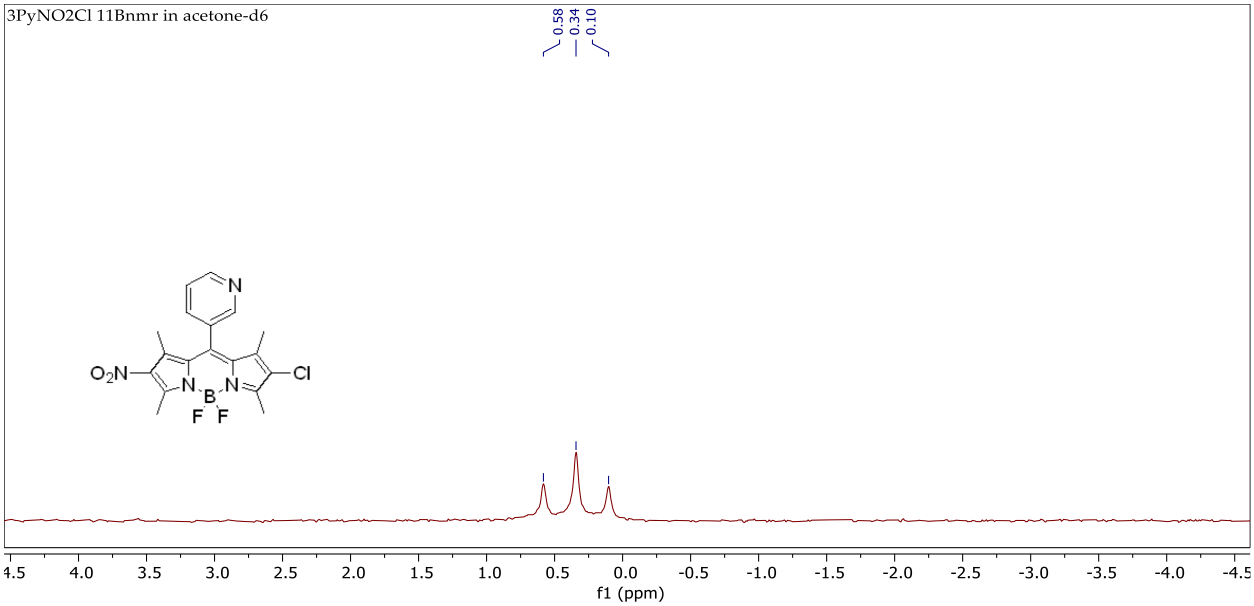

Figure S27. ^{11}B of BODIPY **3PyNO$_2$Cl** in **acetone-d$_6$**

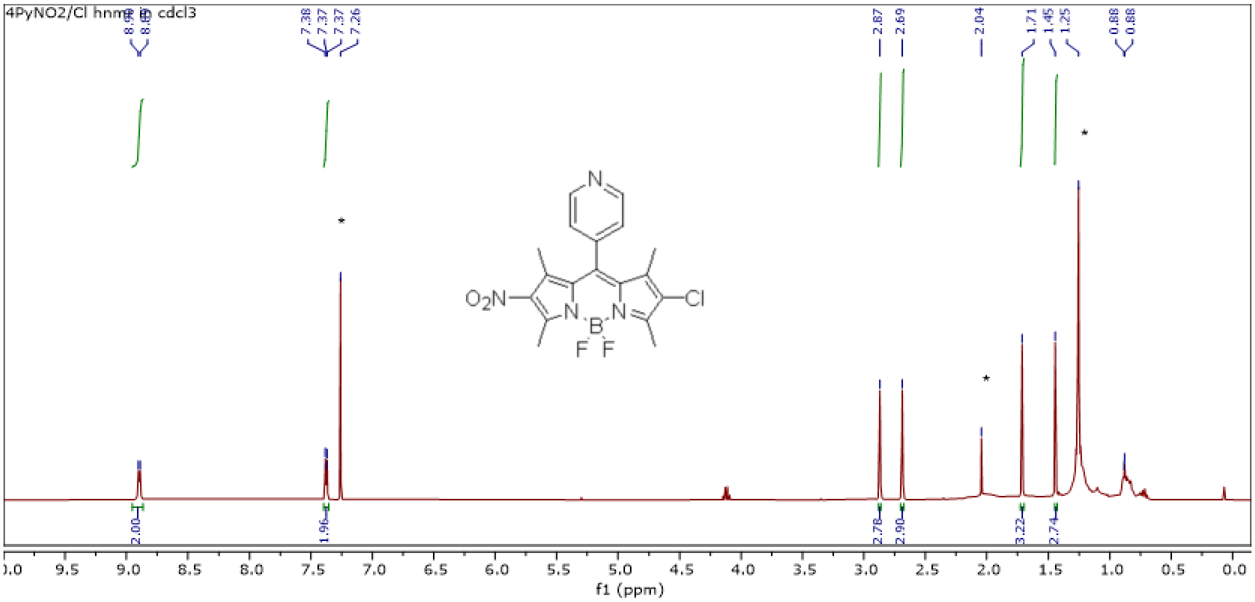

Figure S28. ^{1}H of BODIPY **4PyNO₂Cl** in **CDCl₃**

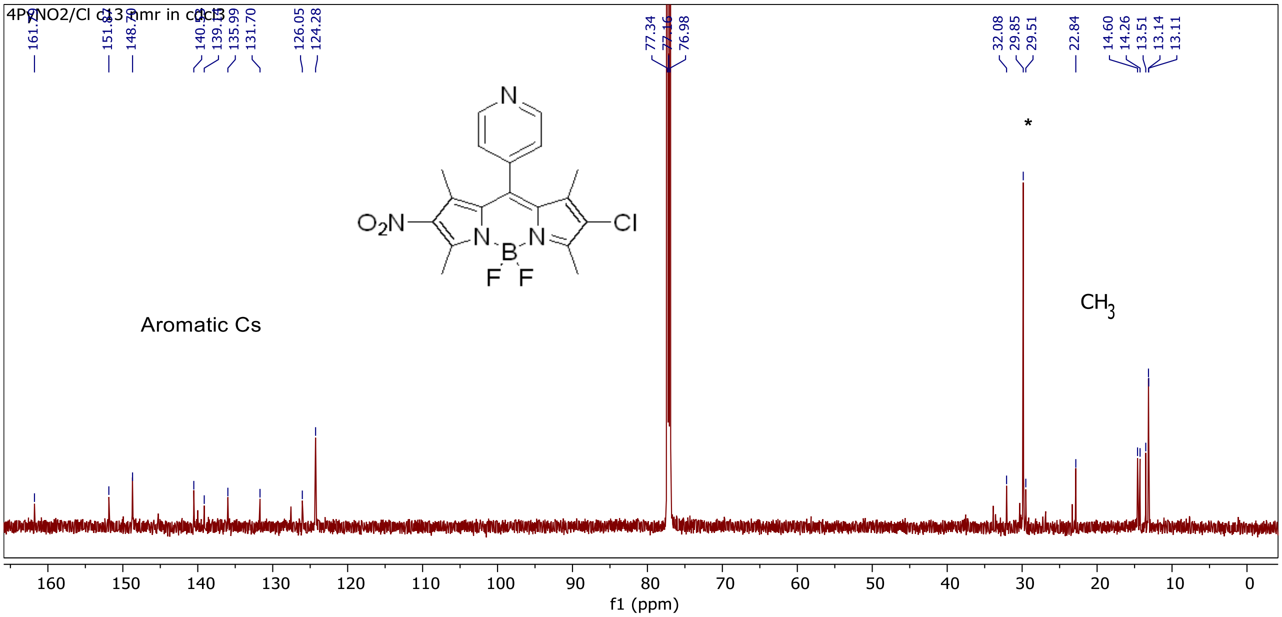

Figure S29. ^{13}C of BODIPY **4PyNO₂Cl** in **CDCl₃**

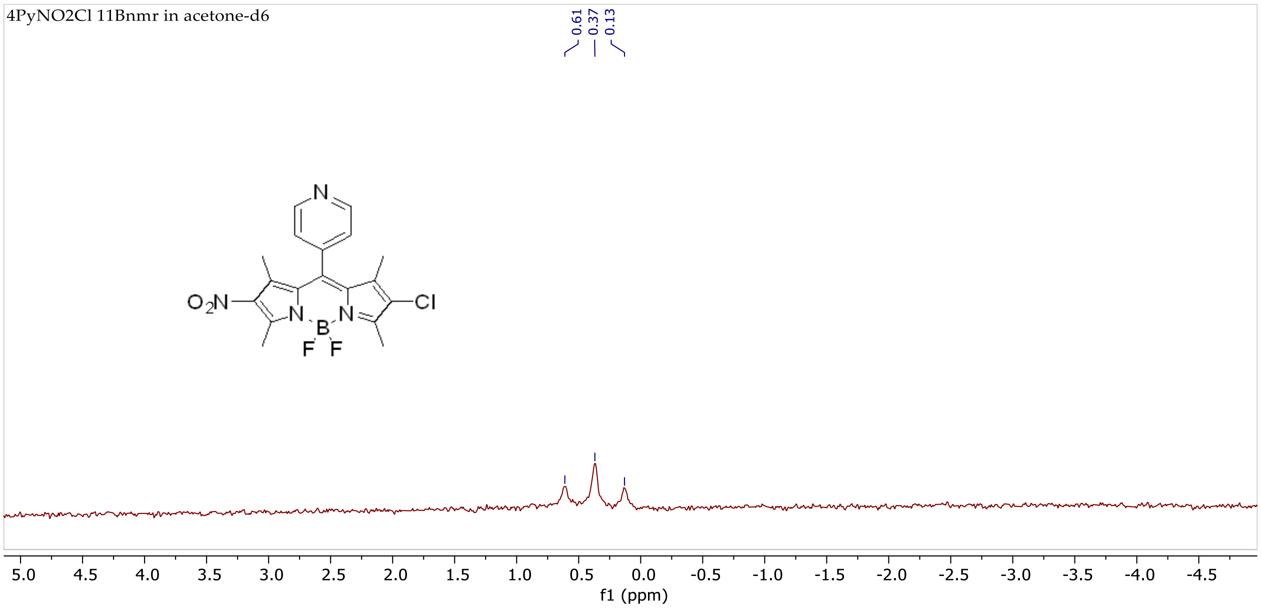

Figure S30. ^{11}B of BODIPY **4PyNO$_2$Cl** in **acetone-d$_6$**
